# Nanoalgosomes: Introducing extracellular vesicles produced by microalgae

**DOI:** 10.1002/jev2.12081

**Published:** 2021-04-27

**Authors:** Giorgia Adamo, David Fierli, Daniele P. Romancino, Sabrina Picciotto, Maria E. Barone, Anita Aranyos, Darja Božič, Svenja Morsbach, Samuele Raccosta, Christopher Stanly, Carolina Paganini, Meiyu Gai, Antonella Cusimano, Vincenzo Martorana, Rosina Noto, Rita Carrotta, Fabio Librizzi, Loredana Randazzo, Rachel Parkes, Umberto Capasso Palmiero, Estella Rao, Angela Paterna, Pamela Santonicola, Ales Iglič, Laura Corcuera, Annamaria Kisslinger, Elia Di Schiavi, Giovanna L. Liguori, Katharina Landfester, Veronika Kralj‐Iglič, Paolo Arosio, Gabriella Pocsfalvi, Nicolas Touzet, Mauro Manno, Antonella Bongiovanni

**Affiliations:** ^1^ Institute for Research and Biomedical Innovation (IRIB) ‐ National Research Council of Italy (CNR) Palermo Italy; ^2^ Centre for Environmental Research Innovation and Sustainability Institute of Technology Sligo Sligo Ireland; ^3^ University of Ljubljana (UL) Ljubljana Slovene; ^4^ Max Planck Institute for Polymer Research (MPIP) Mainz Germany; ^5^ Institute of Biophysics (IBF) ‐ National Research Council of Italy (CNR) Palermo Italy; ^6^ Institute of Biosciences and BioResources (IBBR) ‐ National Research Council of Italy (CNR) Naples Italy; ^7^ Department of Chemistry and Applied Biosciences ETH Zurich Zurich Switzerland; ^8^ Zabala Innovation Consulting Pamplona Spain; ^9^ Institute of Experimental Endocrinology and Oncology (IEOS) ‐ National Research Council of Italy (CNR) Naples Italy; ^10^ Institute of Genetics and Biophysics (IGB) ‐ National Research Council of Italy (CNR) Naples Italy

**Keywords:** biogenic nano‐delivery system, EV‐based therapeutics, extracellular vesicles of non‐mammalian organisms, microalgae, microalgal extracellular vesicles, nanoalgosomes

## Abstract

Cellular, inter‐organismal and cross kingdom communication via extracellular vesicles (EVs) is intensively studied in basic science with high expectation for a large variety of bio‐technological applications. EVs intrinsically possess many attributes of a drug delivery vehicle. Beyond the implications for basic cell biology, academic and industrial interests in EVs have increased in the last few years. Microalgae constitute sustainable and renewable sources of bioactive compounds with a range of sectoral applications, including the formulation of health supplements, cosmetic products and food ingredients. Here we describe a newly discovered subtype of EVs derived from microalgae, which we named nanoalgosomes. We isolated these extracellular nano‐objects from cultures of microalgal strains, including the marine photosynthetic chlorophyte *Tetraselmis chuii*, using differential ultracentrifugation or tangential flow fractionation and focusing on the nanosized small EVs (sEVs). We explore different biochemical and physical properties and we show that nanoalgosomes are efficiently taken up by mammalian cell lines, confirming the cross kingdom communication potential of EVs. This is the first detailed description of such membranous nanovesicles from microalgae. With respect to EVs isolated from other organisms, nanoalgosomes present several advantages in that microalgae are a renewable and sustainable natural source, which could easily be scalable in terms of nanoalgosome production.

## 1 INTRODUCTION

Cells communicate with each other and respond to a variety of stimuli by releasing membrane‐enclosed vesicles, which are found in extracellular fluids (Yáñez‐Mó et al., 2015). Several types of cell‐derived vesicles are commonly distinguished according to their formation mechanism and size. Extracellular vesicles (EVs) have recently emerged as important entities used by cells to mediate several physiological processes or affect various pathological conditions associated with the activation of an immune response or the spread of cancer and virus infections (Dhondt et al., 2020; Maacha et al., 2019; Urbanelli et al., 2019). EVs constitute also cross‐species communication means and have been found in all kingdoms of life (Bleackley et al., 2020; Cai et al., 2018; Gill et al., 2019; Muraca et al., 2015; Soares et al., 2017). Beside mammalian cells, there are various sources available to produce EVs, including bacteria, bovine milk and plants, and indeed several have been studied for therapeutic applications (Bitto & Kaparakis‐Liaskos, 2017; Gerritzen et al., 2017; Kim et al., 2015; Munagala et al., 2016; Paganini et al., 2019; Pocsfalvi et al., 2018; Raimondo et al., 2015; Wang et al., 2013). The exploitation of the biotechnological potential of EVs as carriers of bioactive compounds for different theranostic applications is of increasing interest. The growth of this field is evident from the surge in recent years in the number of publications, patents, companies, and clinical trials related to EVs (Kosaka et al., 2019; Shaimardanova et al., 2020; Zipkin, 2019). As such, in the context of better harmonizing research efforts also aimed at valorising the potential of EVs, Théry and Witwer et al. (2018) recently revised the required parameters for the robust description of EVs (Théry et al., 2018).

Microalgae are microorganisms constituting a rich reservoir of bioactive metabolites such as pigments, polyunsaturated fatty acids, antioxidants or antimicrobial compounds, which are being increasingly exploited in commercial ventures (Cuellar‐Bermudez et al., 2015; Friedl et al., 2021; Khan et al., 2018; Leu & Boussiba, 2014; Zhu, 2015). Microalgae are also heralded as promising feedstock in the context of the bio‐based economy and the better valorisation of natural and renewable bioresources for the production of biofuels, animal feeds and other valuable commodities. This polyphyletic group of microorganisms shows high genetic diversity and has colonised many habitats due to the unique metabolic attributes that some species possess (Friedl et al., 2021). Many microalgae species are suitable for growth in industrial scale photobioreactors under controlled cultivation conditions and are seen as highly productive crops when compared with terrestrial plants (Khan et al., 2018).

In the context of the H2020‐FETOpen project VES4US (www.ves4us.eu) here we propose microalgae as potential bioresources for the production of EVs with applications for the nanomedicine, cosmetics or nutraceutics sectors. In this study, we considered the guidelines of MISEV 2018 and the well‐established knowledge in the EV research field (EV‐TRACK) (Théry et al., 2018; Van Deun et al., 2017) to define a new generation of microalgal EV‐based nanoproducts, using different methodologies and specific approaches for EV bio‐refinement, separation and characterisation. Our check‐list (developed from MISEV guidelines and applied in the framework of the VES4US project) includes the identification of methods for the microalgal‐derived EV separation, enrichment and characterization (including EV quantification, EV identity in terms of protein composition, size, morphology, topology, EV stability, EV quality in terms of purity and density and EV bioactivity), as well as protocols for microalgae cultivation and quantification (Table 1). This list is also reported as supporting information with more details (**Supporting** Table 1), with the purpose to highlight and list the different items we addressed within MISEV 2018. Since the microalgal EVs have to our best knowledge never been described in detail, in accordance with the MISEV 2018 recommendation, we decided to use its suggested nomenclature. Indeed, we refer to the small extracellular particles separated either by differential ultracentrifugation or tangential flow fractionated. The term “nanoalgosome" is here then introduced to describe such microalgal small EVs (sEVs) isolated from the marine photosynthetic microalgal chlorophyte *Tetraselmis chuii*, which is surrounded by a membrane, contains EV biomarkers and has a typical EV size distribution and density (Théry et al., 2006, 2018). *Tetraselmis chuii* is a chlorophyceaen photosynthetic marine microalgae possessing an array of bioactive pigments and essential fatty acids (Pereira et al., 2019), which contribute to making it a promising source of EVs. The production of nanoalgosomes is an evolutionarily conserved trait within the microalgae strain as demonstrated by similar results obtained using the sEVs isolated from batch cultures of other microalgae species, including another chlorophyte strain, the *Dunaliella tertiolecta*, and the dinoflagellate strain *Amphidinium sp*. A drawback limiting progress in current EV research has been the typically low EV yields obtained for subsequent clinical trials, making the roll out of EV‐based treatments for humans still some distance away (György et al., 2015; Paganini et al., 2019). In this context, we envision that microalgae such as *Tetraselmis chuii* can offer a remarkable opportunity to overcome this limitation thanks to their scalable EV production and increased EV yield.

**TABLE 1:** MISEV inspired checklist of minimal information for studies of nanoalgosomes

1. Source features	(a) Microalgal strain, cultivation protocol, and biomass weight (b) Pigment and lipid profiling
2. EV Purification/Enrichment	(a) Differential Ultra Centrifugation (dUC) (b) Tangential Flow Filtration (TFF) (c) gradient Ultra Centrifugation (gUC)
3. EV quantification	(a) Nanoparticle number (by Nanoparticle Tracking Analysis, NTA) (b) Amount of protein (by BCA colorimetric assay)
4. EV identity (size)	(a) Multi angle dynamic light scattering (Multi angle DLS) (b) Nanoparticle tracking analysis (NTA) (c) Fluorescence nanoparticle tracking analysis (F‐NTA) (d) Fluorescence correlation spectroscopy (FCS)
5. EV identity (morphology, shape and membrane presence and structure)	(a) Scanning electron microscopy (SEM) (b) Atomic force microscopy (AFM) (c) Cryogenic transmission electron microscopy (cryo‐TEM) (d) Static light scattering (SLS) (e) Fluorescence nanoparticle tracking analysis (F‐NTA)
6. EV identity (proteins and density)	(a) Immunoblot analysis of EV protein markers (b) Density determination by gUC
7. EV identity (topology)	(a) Fluorescamine assay
8. EV Stability	(a) Zeta potential measurement (b) Stability test/quality control in biological fluids (c) Resistance to detergents
9. EV Bioactivity	(a) In vitro cytotoxicity (b) Cellular uptake
10. Quality control (purity)	(a) Negative control on EV identity and preparation on culture media – throughout the manuscript (b) EV quantification by labelling with fluorescent lipid specific dye, to assess the presence of non‐EV particles – see also 5.e (c) Density measurement and separation by density gradient, to assess the ratio between EVs and aggregates – see also 6b (d) Application of a tailor‐made Quality Management System (QMS)

## 2 MATERIALS AND METHODS

### 2.1 Microalgae cultivation

A stock culture of the marine chlorophyte *Tetraselmis chuii* CCAP 66/21b was grown in borosilicate glass vessels in f/2 medium (Guillard, 1975) into its exponential phase growth and used via a 10% v/v inoculum to start 50 ml batch‐cultures, in glass tubes, or 7.5 litre cultures, in a photobioreactor PB 200 (GroTech GmbH, Germany), at an initial concentration of 0.5 mg/ml (wet weight). Tubes and reactor were maintained at a temperature of 20 ± 2°C, a white light intensity of 100 μE m^−2^ s^−1^ and 14:10 light/dark photoperiod for 30 days prior to processing by EVs separation. Aeration was provided using a 0.22 μm airline and homogenisation was carried out manually every 3–4 days. The same batch cultivation procedures were used for other two microalgal strains: *Dunaliella tertiolecta* and the *Amphidinium sp*.

An aliquot of the biomass of microalgal cells was collected at day 30 by centrifugation (2000 × *g* 10 min) and freeze‐dried overnight prior to weight or storage at ‐20°C. The biomass was treated with 1 ml of 0.5 M ammonium formate for desalting prior to freeze‐drying.

#### 2.1.1 Pigment extraction and analysis

Pigment extraction was carried out according to Mc Gee et al. (2018). Samples of freeze‐dried biomass (2‐3 mg) were mixed with 500 μl of ice cold 100% acetone and glass beads and placed in a FastPrep FP120 ribolyser for 40 s at full speed. Deionised water was added to bring the solution to 80% acetone (v/v) and vortexed. The extracts were then filtered through 0.22 μm PTFE membrane syringe filters to remove any residual particulate material. The extracts were transferred into amber vials and stored at ‐80°C and analyzed within 24 h. Pigment extracts were analysed at constant room temperature on a Varian ProStar HPLC binary solvent delivery system equipped with a 20 μl sample loop, ProStar 310 UV and 335 PDA detectors. Pigments were separated using a Phenomenex Onyx C18 100 × 4.6 mm ID monolithic column fitted with a Phenomenex Onyx C18 guard cartridge 10 × 4.6 mm ID employing a stepped gradient solvent programme with a flow rate of 3 ml/min. Pigments were resolved using a gradient profile consisting of 10% B starting condition for 0:10 min, followed by a linear gradient to 65% B from 0:10–2:00 min, isocratic hold at 65% B from 2:00 to 4:00 min, linear gradient from 4:00 to 5:00 min followed by hold at 90 B for 1:00 min and a final re‐equilibration at initial conditions from 6:01–7:50 min. The mobile phase A consisted of methanol: 0.5 M ammonium acetate (80:20 v/v) and mobile phase B was acetone: acetonitrile (70:30 v/v). Prior to injection, extracts were diluted (1:5) with 0.5 M ammonium acetate when necessary. Carotenoids and chlorophylls were detected with a diode‐array detector, scanning absorbance spectra from 360 to 700 nm and monitoring at 450 nm for optimal carotenoid detection. Probable pigment identification was achieved by comparing retention times and UV‐vis spectral fine structures to pigment standards, DHI phytoplankton pigment Mix‐115 and reference data‐sheets.

#### 2.1.2 Lipid extraction and fame analysis

The freeze‐dried microalgal biomass was extracted according to Ryckebosch et al. (2012) with slight modifications. First, 400 μl of methanol was added to dried biomass (2‐15 mg), followed with 200 μl of chloroform and 40 μl of deionised water. The sample was then vortexed and centrifuged (2,000 rpm, 10 min). The supernatant was discarded and the bottom chloroform layer collected. The residual biomass in the tube was re‐extracted using 200 μl of methanol and chloroform, vortexed and centrifuged again. The upper layer was collected, and the extraction was carried out twice more on the residual biomass. The four lipid extract layers were then pooled together into a 15 ml tube and Na_2_SO_4_ salts added for dewatering. Upon further centrifugation, the solution was placed in a new tube and the sample was then evaporated to dryness under a nitrogen stream. The residue was then resuspended in 500 μl of chloroform:methanol (50:50) as the final extract. Prior to analysis, 200 μl of the sample was placed in a GC‐MS vial fitted with a glass insert and supplemented with 50 μl of trimethylsulfonium hydroxide (TMSH) for transesterification. The samples were left for at least 1 h at room temperature prior to analysis by GC‐MS. The separation of Fatty Acid Methyl Esters (FAMEs) in the microalgal extracts was carried out using a BPX70 120 m column with an internal diameter of 0.25 mm on an Agilent7890A/5975C GC‐MS system equipped with the MassHunter software. Samples were injected at a split ratio of 100:1 at an inlet temperature of 250°C with the helium flow rate set at 2 ml/min (48.51 psi) and the transfer line at 280°C. The oven gradient temperature was as follows: an initial hold at 50°C for 2 min followed by 20°C/min ramp to 160°C for 0 min, a 4°C/min ramp to 220°C for 5 min and finally a 4°C/min ramp to 240°C for 12.5 min. The mass spectrometry conditions had a solvent delay of 10.5 min. Identifications were carried out by comparing retention times against standards of the Supelco 37 Component FAME Mix and using the MS NIST 08 library.

### 2.2 Separation of nanoalgosomes from microalgae‐conditioned media

#### 2.2.1 Centrifugation‐based EV purification methods: differential centrifugation

The 50 ml batch‐cultures were centrifuged on day 30 at low speed to separate cells from the culture medium. Then, the separation of microalgae‐derived EVs was performed by differential centrifugation (dUC) (Romancino et al., 2013). Large EVs (lEVs) were isolated in 50 ml Eppendorf polypropylene conical tubes at 10,000 × g for 30 min at 4°C using an Eppendorf rotor F34‐6‐38. Small EV (sEVs) were then collected from the supernatant into Beckman Coulter polypropylene open top tubes via centrifugation at 118,000 × g for 70 min at 4°C using a Beckman SW28 rotor. After a Phosphate‐buffered saline (PBS 1X, without Calcium and magnesium, Thermo Fisher Scientific) washing step, the pellet was re‐suspended in PBS for subsequent analyses, these sEV preparations are the dUC‐isolated nanoalgosomes.

#### 2.2.2 Filtration‐based EV purification methods: Tangential flow filtration

Cell clarification and EV concentration were performed using a TFF ÄKTA Crossflow system (GE Healthcare, USA) and three GE Healthcare polysulfone hollow fibre membranes. After 30 days of cultivation, the reactor (containing typically 7.5 L of cell culture) was connected to the TFF system and the cell suspension was clarified by microfiltration with a 450 nm hollow fibre cartridge housed in the ÄKTA Crossflow. Feed flow and transmembrane pressure (TMP) were kept constant at 110 ml/min and 0.05 bar, respectively. The first retentate (> 450 nm sized particles) was concentrated into a final volume of 100–200 ml and used to observe by light microscopy the integrity of cells. The 450 nm permeate (< 450 nm sized particles) was processed for a second microfiltration step using a 200 nm hollow fibre membrane with a 140 ml/min feed flow and 0.05 bar TMP. The ensuing permeate (< 200 nm sized particles) was concentrated using a 50 kDa MWCO hollow fibre membrane with feed flow and TMP of 42 ml/min and 0.45 bar, respectively; these TFF preparations corresponds to the sEVs and are considered as the TFF‐isolated nanoalgosomes.

#### 2.2.3 Density‐based EV purification methods: gradient ultracentrifugation

Gradient ultracentrifugation was used to further purify selected samples enriched in sEVs either by TFF or dUC. Samples containing about 200 μg of sEVs (expressed in protein content and measured by BCA) were concentrated by ultracentrifugation at 110,000 × *g* for 2 h at 4°C using a Ti70 rotor. The resulting pellets were homogenized in 10 mM Tris‐HCL pH 8.6 buffer in a final volume of 50 μl. Samples were vigorously mixed for 20 min to disaggregate vesicles. A 50% (w/v) iodixanol working solution was prepared by diluting OptiPrep (Merck) according to the manufacturer's instruction. A discontinuous gradient containing 1.5 ml of 50%, 30% and 10% gradient cushions was prepared and the samples containing the EVs of interest were layered on top of the gradient. Ultracentrifugation was carried out at 110,000 x *g* for 24 h at 4°C using an SW55Ti rotor. Ten fractions of 500 μl each were collected from the top of the tubes. The percentage of iodixanol in each fraction was measured using a UV spectrophotometer (Nanodrop 200, Thermo Scientific) at 340 nm, from which the density was calculated according to the manufacturer's method. Protein concentration in each fraction was measured by micro‐BCA (Thermo Scientific) using a UV spectrophotometer (Nanodrop 200, Thermo Scientific) at 562 nm.

### 2.3 Characterisation of nanoalgosomes

#### 2.3.1 BCA assay and immunoblotting

The protein content of microalgal EVs was measured using the micro‐bicinchoninic BCA Protein Assay Kit (Thermo Fishers Scientific). This colorimetric method provides a relative concentration to a protein standard (bovine serum albumin, BSA), which is used for the preparation of a calibration curve. The relative absorbance of the BCA soluble compound was measured at 562 nm using a GloMax Discover Microplate Reader. Proteins were separated by sodium dodecyl‐sulfate polyacrylamide gel electrophoresis (SDS‐PAGE) (10%). A total of 30 μg of cell lysate and EV samples (in PBS) were mixed with proper volumes of 5X loading buffer (0.25 M Tris‐Cl pH 6.8, 10% SDS, 50% glycerol, 0.25 M dithiothreitol (DTT), 0.25% bromophenol blue). Then, the samples were heated at 100°C for 5 min and loaded in a 10% sodium dodecyl sulfate‐polyacrylamide gel for electrophoretic analyses. Proteins were blotted onto polivinilidenfluoro‐membranes (PVDF), which were blocked with BSA‐TBS‐T solution (3% powdered with bovine serum albumin in TBST (50 mM Tris HCl pH 8.0, 150 mM NaCl with 0.05% Tween 20) for 1 h at room temperature, followed by primary antibody incubation overnight at 4°C. The antibodies anti‐Alix (clone 3A9, dil. 1:150 in 3%BSA/TBS‐T1X), anti‐Enolase (clone A5, dil. 1:400 in 3%BSA/TBS‐T1X), anti‐β‐actin (clone AC15 dil. 1:400 in 3%BSA/TBS‐T1X) and anti‐HSP70 (clone W27 dil. 1:500 in 5% Milk/TBS‐T1X) (Santa Cruz Biotechnology, USA), raised against different mammalian EV markers (MISEV 2018), also showed cross‐reactivity to microalgae and were used in the present study. Anti H+/ATPase (dil. 1:1000 in 3% BSA/TBS‐T1x, Agrisera), with a predicted reactivity for microalgae, are incubated for 1 h at room temperature. After washing, the membranes were incubated for 1 h with secondary antibodies according to the manufacturer's instructions (horseradish peroxidase‐conjugated secondary anti‐mouse or anti‐rabbit antibodies, Cell Signaling). The membranes were washed four times in TBST for 20 min. The immunoblots were revealed using SuperSignal Pierce ECL (Thermo Fisher Scientific).

#### 2.3.2 Nanoparticle tracking analysis

Nanoparticle size distribution and concentration were measured using a NanoSight NS300 (Malvern Panalytical, UK) at CNR and a ZetaView instrument (Particle Metrix) at ETH Zurich. The first instrument was equipped with a 488 nm laser, a high sensitivity sCMOS camera and a syringe pump and the second a 405 laser and a CMOS camera. At CNR, EV samples were diluted in particle‐free water (Water, HPLC grade, Sigma‐Aldrich, filtered by 20 nm using Whatman Anotop filters) to generate a dilution in which 20–120 particles per frame were tracked, to obtain a concentration within the recommended measurement range (1–10  ×  10^8^ particles/ml). Five experiment videos of 60‐s duration were analyzed using NTA 3.4 Build 3.4.003 (camera level 15–16). A total of 1500 frames were examined per sample, which were captured and analysed by applying instrument‐optimized settings with a suitable detection threshold so that the observed particles are marked with red crosses and that no more than 5 blue crosses are seen. Further settings, such as blur size and Max Jump Distance were set to ‘automatic’ and viscosity to that of water (0.841 ‐ 0.844 cP). At ETH Zurich, the EV samples were diluted in particle‐free PBS to obtain particle concentrations between 10^7^ and 10^9^ particles/ml. Each sample was injected with a 1 ml syringe in the sample chamber which was calibrated daily with polystyrene nanoparticles. Videos were acquired at 11 positions in the chamber at a frame rate of 30/s, with a trace length of 15 frames and using 80% scattering intensity and 150 shutter in light scattering mode. The experiments were repeated in triplicates and analysed with the ZetaView analysis software (ZetaView 8.04.02 SP2).

#### 2.3.3 Fluorescence nanoparticle tracking analysis (F‐NTA)

We explored several staining strategies including commercial apolar dyes for lipid membrane staining (*e.g*., DiI and DIOC16) and photoactivatable probes to stain apolar environments (e.g. Di‐8‐ANEPPS). Therefore, we labelled nanoalgosomes with the dye Di‐8‐ANEPPS, whose fluorescence is activated in apolar environments, and specifically enhanced when bound to the lipid membrane of EVs, with a higher quantum yield with respect to any binding to hydrophobic protein regions. This makes it the Di‐8‐ANEPPS fluorescent signal highly EV‐specific. For F‐NTA, the fluorescent *Tetraselmis chuii*‐derived EVs (f‐EVs) were produced as follows: 5 × 10^10^ EV particles/ml were stained with 500 nM of 4‐(2‐[6‐(dioctylamino)‐2‐naphthalenyl]ethenyl)‐1‐(3‐sulfopropyl)pyridinium, DI‐8‐ANEPPS (Ex/Em: 467/631 nm, ThermoFisher Scientific), previously filtered by 20 nm filters (Whatmann Anotop filters). After 1 h at room temperature, NTA analyses were carried out by using NanoSight NS300 (Malvern Panalytical, UK) with a 500LP filter (laser wavelength 488 nm), with optimized manual settings for camera level and with high flow rate for the syringe pump (setting 150 μl/s) so that fluorescent EVs (f‐EVs) cross the field of view of the main NTA screen in 5 to 10 s. Further settings were set as described in the previous NTA section. As negative control, we tested that the probe alone does not emit fluorescence signal with F‐NTA.

#### 2.3.4 Fluorescence correlation spectroscopy (FCS)

FCS experiments were performed on nanoalgosomes labelled with Di‐8‐ANEPPS by using a Hamamatsu C9413‐01 instrument equipped with a 473 nm excitation source. Along with the samples, a 10 nM Alexa‐488 solution was used in the multi‐well glass container as a calibrant for concentration and characteristic diffusion times to optimize the optical setup (Montis et al., 2017; Pánek et al., 2018; Ries & Schwille, 2012). In the Supporting Information, further details are reported on the experimental set up and the analysis to obtain the size distribution function.

#### 2.3.5 Multi angle dynamic light scattering (DLS)

Multi angle DLS experiments were repeated at two different laboratories of our consortium, CNR (Italy) and MPIP (Germany), by using a slightly different procedure. Briefly, samples were diluted to a final total protein content below 50 μg/ml to avoid vesicle interaction and multiple scattering, and then either centrifuged at 1000 × g for 10 min and poured into a quartz cylindrical cell or directly filtered into the cell by Millex‐LCR 0.45 μm syringe filters (Merck, Germany) at CNR or MPIP, respectively. At CNR, cells were placed at 20°C in a thermostated cell compartment of a Brookhaven Instrument BI200‐SM goniometer equipped with a solid state laser tuned at a wavelength ***λ *** = 532 nm, and a Brookhaven BI‐9000 correlator (Brookhaven Instruments, Holtsville, NY, USA). At MPIP, multi‐angle DLS was performed with an ALV spectrometer (ALV‐GmbH, Germany), including a goniometer and an ALV/ LSE‐5004 multiple‐tau full‐digital correlator with 320 channels and equipped with a He‐Ne laser with ***λ *** = 632.8 nm. Scattered intensity and intensity autocorrelation function *g_2_(t)* were measured simultaneously at different scattering angle ***ϑ*** to measure the z‐averaged hydrodynamic diameters *D_h0_*, the average diameter *D_g_*, which is twice the radius of gyration, and eventually the full size distribution (Mailer et al., 2015; Noto et al., 2012; Prima et al., 2020; Schmitz, 1990). Full details about the analysis are reported in the Supporting Materials and Method's Section and **Supporting Figure S1**.

Analogous DLS experiments were performed at different pHs. The TFF‐separated sEVs were pelleted by ultracentrifugation and then resuspended in a 10 mM buffer solution with 150 mM NaCl. The buffers used were phosphate buffer (pH 6.1, 7.4 and 7.8), acetate buffer (pH 4.3 and 5.3), or carbonate buffer (pH 8.8).

DLS experiments with detergents were performed at the same vesicle concentration using different detergents concentrations. Samples were incubated overnight, centrifuged at 1000 × *g* for 10 min and supernatants poured in quartz cells for DLS measurements. Both SDS and Triton X‐100 were purchased from Sigma Aldrich, diluted in buffer solution and filtered using 200 nm syringe filters. For comparison the same experiments were performed on large unilamellar vesicles (LUV) made with 2‐Oleoyl‐1‐palmitoyl‐sn‐glycero‐3‐phosphocholine (POPC), 2‐Oleoyl‐1‐palmitoyl‐sn‐glycero‐3‐phospho‐L‐serine sodium salt (POPS), both purchased from Avanti Polar Lipids Inc. (Alabaster, AL, USA), and Cholesterol (Merck Life Science). LUV were made with the relative content of POPC:POPS:Cholesterol (81:9:10) by extrusion through a 100 nm polycarbonate filter by using a miniextruder (AVESTIN, Germany).

#### 2.3.6 Atomic force microscopy (AFM)

Atomic Force Microscopy images were captured by using a Nanowizard III scanning probe microscope (JPK instruments, AG Germany) equipped with a 12 μm scanner. Nanoalgosomes were initially concentrated by ultracentrifugation and resuspended in MilliQ water to a final concentration of 5 × 10^11^ particles/ml, as previously estimated by NTA.

For measurements on dry samples, a 30 μl drop of the samples was directly deposited on freshly cleaved mica, incubated for 10 min, and then gently dried under nitrogen flow. Measurements were performed in tapping mode by using a NSC‐15 (Mikromasch) cantilever (spring constant 40 N/m, typical tip radius 8 nm). Measurements with softer cantilevers (data not shown) were carried out with MSNL‐10 cantilevers (Bruker; lever D, spring constant 0.03 N/m, nominal tip radius 2 nm).

#### 2.3.7 Scanning electronic microscopy

Samples were fixed in 0.4% paraformaldehyde and 2.5% glutaraldehyde in 300 mM PBS at 4°C. The pre‐fixed samples were applied onto polycarbonate filters with pore‐diameter of 0.05 μm (STERLITECH) until the filter got blocked. Then, the EV‐covered filters were taken from holders and post‐fixed in a bath following the protocol adopted from (Lešer et al. (2009). In brief, the primary fixatives were removed by three washing steps with distilled water (10 min incubation for each). Samples were then incubated for 1 h in 2% OsO4. They were washed with distilled water (three washing steps with 10 min incubation time), treated with saturated water solution of thiocarbohydrazide (15 min incubation time), washed again (three washing steps in distilled water, 10 min incubation time each), and subjected to 2% OsO4 again for 1 h. After the second incubation in OsO4, the unbound osmium was removed in three additional washing steps (in distilled water, 10 min incubation time in each step). The samples were dehydrated in graded series of ethanol (30%‐100%, 10 min incubation in each solution; absolute ethanol was replaced three times), followed by graded series of HMDS (mixed with absolute ethanol; 30%, 50% and 100%, 10 min in each solution) and finally air dried. The dried samples were Au/Pd coated (PECS Gatan 682) and examined by JSM‐6500F Field Emission Scanning Electron Microscope (JEOL Ltd., Tokyo, Japan).

#### 2.3.8 Cryo‐transmission electron microscopy (cryo‐TEM)

The sEV samples, with an original particle concentration of 5 × 10^10^ per ml, were concentrated 10x (using Amicon Ultra‐2 μl Centrifugal Filters, Molecular weight cut‐off (MWCO): 30 kDa, Merck, Germany) and afterwards 10 μl were placed onto a 400 mesh copper grid covered with lacey film, which was treated with oxygen plasma to make it hydrophilic, and immobilized using high pressure freezing (Engineering Office M. Wohlwend GmbH, Switzerland). The specimen (sapphire discs with cells) was enclosed and protected in a small volume between two specimen carriers and locked inside the specimen pressure chamber by blotting two times for 3 s each. Liquid nitrogen was used as cooling medium. After the preparation, the samples were carefully transferred into liquid nitrogen for further imaging. Imaging was performed on a TEM (FEI Tecnai F20) microscope. For the Cryo‐TEM analysis, the acceleration voltage was 200 kV and the device was coupled with an axis Gatan US1000 2k CCD camera.

#### 2.3.9 Stability and surface properties: zeta‐potential and amino‐groups of nanoalgosome surface

A Zetasizer Nano Z (Malvern Panalytical GmbH, Germany) with disposable folded capillary cells was used to determine the zeta‐potential (ζ‐potential) of the nanoalgosomes. Basically, 50 μl of each sample were diluted with 1 μl of a 1 mM potassium chloride (KCl) solution. The measurement was performed at 25°C after 2 min of equilibration. Each measurement was repeated in triplicate and mean values, as well as standard deviations, were calculated.

The amount of NH_2_ groups present on the nanoalgosome surface was determined based on a fluorescamine assay (FA assay). Hexylamine, which contains primary amine groups, was selected as a reference for establishing the standard calibration curve. For the assay, 250 μl fluorescamine stock solution (concentration of 0.3 mg/μl) and 25 μl sample solution (H_2_O as the control), as well as 725 μl borate buffer (0.1 M, pH = 9.5), were added into a 2 μl Eppendorf tube. The mixture was vortexed (Heidolph REAX2000 at maximum speed) for 30 s and then immediately analyzed in a plate reader (Tecan AG, Switzerland) at 25°C by exciting at 410 nm and detecting the fluorescence emission at 470 nm. Samples were concentrated 10x before the quantification using Amicon Ultra‐2 μl Centrifugal Filters, Molecular weight cut‐off (MWCO): 30 kDa, Merck, Germany. All fluorescence measurements were repeated three times (3 × 100 μl in a well of a 96‐well‐plate).

#### 2.3.10 Stability in biological fluids

The stability of nanoalgosomes was analysed in undiluted human blood plasma using multiangle dynamic light scattering. Human citrate blood plasma was collected from 10 healthy donors at the Transfusion Centre of the University Clinic of Mainz, Germany, according to standard guidelines. It was pooled and stored at ‐20°C. To remove cell fragments and additional protein precipitates, it was centrifuged at 20,000 × g and 4°C for 1 h (Sigma 3–30K, Germany) before use.

The nanoalgosome samples were centrifuged at 10,000 × g for 10 min prior to analysis to remove dust. The citrate plasma (200 μl) was filtered through 0.2 μm Millex‐GS syringe filters (Merck, Germany) directly into cylindrical quartz cuvettes (18 mm diameter, Hellma, Germany). The cuvettes were cleaned in an acetone fountain prior to usage for removing dust. Then, 10 μl of *Tetraselmis chuii*‐derived nanoalgosomes were added. For reference, 10 μl of pure nanoalgosomes were diluted in 200 μl PBS buffer. Similarly, 200 μl of plasma were measured without the addition of nanoalgosomes. Samples were incubated at 37°C for 1 h before the measurement.

Multi‐angle DLS was performed with an ALV spectrometer (ALV‐GmbH, Germany) at 37°C. The set‐up consisted of a goniometer and an ALV/LSE‐5004 multiple‐tau full‐digital correlator with 320 channels. As a light source, a He‐Ne laser was used at a wavelength of 632.8 nm. Data analysis was performed using a multicomponent fitting method according to Rausch et al. (2010).

### 2.4 Bioactivity and cellular uptake of nanoalgosomes

#### 2.4.1 Cell cultures

The following cell lines were used for the bioactivity and cellular uptake analyses: (i) 1–7 HB2, a normal mammary epithelial; (ii) MDA‐MB 231, an epithelial human breast cancer and (iii) HepG2 a human hepatocarcinoma (ECACC cell lines). All cell lines were maintained at 37°C in a humidified atmosphere (5% CO_2_) in Dulbecco's Modified Eagle's Medium (DMEM) (Sigma‐Aldrich) containing 10% (v/v) Fetal Bovine Serum (FBS) (Gibco, Life Technologies) plus 2 mM L‐glutamine, 100 U/ml Penicillin and 100 mg/ml Streptomycin (Sigma‐Aldrich) for the MDA‐MB‐231; DMEM low glucose plus 5 μg/ml Hydrocortisone and 10 μg/ml, Bovine Insulin (Sigma‐Aldrich) for 1–7 HB2 cell line; RPMI 1640 Medium containing 10% (v/v) Fetal Bovine plus mM L‐glutamine, 100 U/ml Penicillin and 100 mg/ml Streptomycin was used for HepG2 cell line.

#### 2.4.2 Cell viability assay

Tumoral (MDA‐MB 231 and HepG2) and normal (1‐7 HB2) cell lines were seeded in 96‐well plates at a density of 2 × 10^3^ cells per well and maintained using suitable culture conditions. The assay was carried out with EVs isolated from *Tetraselmis chuii* conditioned media. Similar to other studies carried out with plant‐derived EVs, the nanoalgosomes were used at concentrations ranging from 0.1 to 2.0 μg/ml (Kim et al., 2015; Montis et al., 2017; Raimondo et al., 2015). This is equivalent to ∼10^4^‐10^5^ EVs/cell, corresponding to the estimated number of vesicles considered necessary to cover the surface of a cell (Sverdlov, 2012). 24 h after seeding, the cells were incubated for 24, 48 and 72 h with *Tetraselmis chuii*‐derived EVs. The cells treated with PBS alone were used as control. Cell viability was evaluated using the CellTiter 96 AQueous One Solution Reagent (Promega) according to the manufacturer's instructions. The mean optical density (OD, absorbance) of four wells in the indicated groups was used to calculate the percentage of cell viability as follows: percentage of cell viability = (A_treatment_ − A_blank_)/(A_control_ (untreated) − A_blank_) × 100% (where, A = absorbance_490nm_). Values were expressed as means ± SD of three biological samples, each performed in triplicate.

#### 2.4.3 Genotoxicity assay

MDA‐MB 231, HepG2 and 1–7 HB2 cells were plated in 24‐well plates containing sterile coverslips in complete medium, for 24 h. Cells were then incubated with 2 μg/ml of *Tetraselmis chuii*‐derived EVs for 48 and 72 h. Thereafter, the medium was removed and the cells were washed twice with PBS and subsequently stained with Acridine Orange/PBS solution (Sigma) at 100 μg/ml for 10 s at room temperature and quickly examined by epi‐fluorescence microscopy (Leica, DFC450C). Acridine Orange is a cell permeating nucleic acid binding dye that emits green fluorescence when bound to double‐strand DNA and red fluorescence when bound to single‐strand DNA, RNA or lysosomes. This staining technique allows for discrimination between intact (green nuclei) and damaged DNA in cells (red nuclei).

#### 2.4.4 Cellular uptake

MDA‐MB 231 and 1–7 HB2 cell lines were grown at a density of 5 × 10^3^ cells/well in 12‐well plates containing sterile coverslips in complete medium for 24 h. F‐EV samples (see F‐NTA section), were dialysed to remove free probe (D‐Tube Dialyzer Midi, MWCO 3.5 KDa, Sigma‐Aldrich) against PBS, for 24 h at 4°C. As negative control, we used dUC isolates (at 118,000 × g) using not‐conditioned f/2 media incubated with Di‐8‐ANEPPS, similarly to f‐EV samples. Before and after dialysis, both f‐EVs and negative control were checked for fluorescence and size distribution by NTA. Next, cell lines were incubated with different amount of f‐EVs (0.5 and 2 μg/ml) at 37°C or 4°C, as well as with an equivalent amount of negative control. After different incubation times (3, 6 and 24 h), cells were washed twice with PBS, fixed with 3.7% paraformaldehyde for 15 min, and washed again twice with PBS. F‐actin cytoskeleton was detected by staining with Alexa Fluor 594 phalloidin (ThermoFisher Scientific) in 1% BSA‐PBS solution at 400 μgr/ml for 60 min at room temperature. Afterwards, the nuclei were stained with VECTASHIELD Mounting Medium with DAPI. The f‐EV cellular localization was monitored by fluorescence microscopy analysis (Nikon Eclipse 80i), confocal laser scanning microscopy (Olympus FV10i, 1 μm thickness optical section was taken on total of about 15 sections for each sample) and analysed using ImageJ 1.52t. The reported relative green fluorescence intensities correspond to the fluorescence intensities of 5 different images of each sample, normalized to the number of nuclei, and relative to the control sample (i.e., cells treated with the dUC‐isolates from not‐conditioned f/2 media). Experiments were repeated using three independent nanoalgosome preparations; data were expressed as the mean ± standard deviation (SDs).

#### 2.4.5 Statistical analyses

Experiments were independently repeated at least in triplicate. Error bars in the graphical data represent standard deviations. A Student's t‐test was used for statistical analysis, and statistical significance was claimed when the *P*‐values were ≤ 0.0001 (****) and ≤ 0.001 (***).

### 2.5 Quality management system

We validate and applied a Quality Management System (QMS) compatible with UNI EN ISO 9001:2015 standard to assure standardization of the procedures as well as reliability and reproducibility of the results among the different laboratories. Our QMS supported the scientific activities of the study, including the sharing of standard operating procedures (SOPs) to increase the reliability and reproducibility of the results. Customized lab notebooks and SOP models were developed, distributed and utilized among the participating laboratories. Quality Assurance and Quality Control activities, including checklists and review meetings, were performed to monitor the specific activities of partners (Liguori & Kisslinger, 2020).

### 2.6 EV‐track

We have submitted all relevant data of our experiments to the (EV‐TRACK ID: EV200075) (Van Deun et al., 2017).

## 3 RESULTS

The following paragraphs describe an in depth biochemical, biophysical and biological characterization of the microalgal‐derived small extracellular vesicles, sEVs, which we named nanoalgosomes.

### 3.1 Characterization of the nanoalgosome bioresource: *Tetraselmis chuii*

The marine photosynthetic chlorophyte species *Tetraselmis chuii* CCAP66/21b was identified in the present study as a new source of nanoalgosomes. First, we determined the chemical signatures of the microalgal biomass in terms of pigment and fatty acid methyl ester (FAME) contents. Interestingly, we found high‐value carotenoid (*e.g*., Neoxanthin, Violaxanthin, Lutein, and β carotene) and long‐chain polyunsaturated fatty acids (LC‐PUFA), such as the eicosapentaenoic (EPA, 20:5n‐3) (**Supporting Figure S2**).

### 3.2 Separation and quantification of microalgal extracellular vesicles

Next, from the microalgae‐conditioned culture media we isolated extracellular by‐products, which consist of two EVs sub‐populations: nanovesicles (small EVs, sEVs) and microvesicles (large EVs, lEVs). While differential ultracentrifugation (dUC) is the classical method for EV enrichment (Romancino et al., 2013; Théry et al., 2006), tangential flow filtration (TFF) has been increasingly applied in the field, as gentler (low shear stress) purification method to optimise the recovery of intact EVs with consistent purity both in small and large‐scale processes (Busatto et al., 2018; Paganini et al., 2019).

Both dUC and TFF procedures allowed a reproducible separation of sEVs; the yield of sEV production was slightly affected by the scaling‐up of the microalgal culture from small‐ (50 ml) to medium‐scale (7.5 L) and the use of two different separation protocols (Table 2). The EV yields, in terms of sEV protein content and sEV number, were consistent with the estimate of 10^9^ EV particles/μg EV proteins, as reported by Sverdlov (2012) (Sverdlov, 2012). Based on the above analyses, we have validated and optimized the TFF separation method and were able to produce sufficient amount of sEVs for the in‐depth characterization, in line with those obtained from plant‐derived vesicles, as reported for citrus juice‐derived nanovesicles (Raimondo et al., 2015).

**TABLE 2:** yield of nano‐algosomes (microalgal‐derived sEVs) from six biological replicates of *Tetraselmis chuii*‐conditioned media from small‐ (50 ml) to medium‐scale (7.5 L) volume

Isolation method	Culture Volume	sEV pt μg/mg biomass	n. sEVs/mg biomass
dUC (118,000 × *g* pellet)	50 ml	0.40 ± 0.03	(2.00 ± 0.08) × 10^9^
TFF (< 200 nm)	7500 ml	0.30 ± 0.05	(1.00 ± 0.20) × 10^9^

The sEV yields is based on EV protein concentration (sEV pt), measured by micro‐bicinchoninic (BCA) colorimetric assay, and particle numbers (n. sEVs), measured by Nanoparticle Tracking Analysis (NTA), both expressed per mg of dry weight microalgal biomass.

### 3.3 Nanoalgosome identity: biophysical analyses of particle size

Microalgal sEVs, *i.e*. nanoalgosomes, were analyzed by different techniques for determination of their average size and size distributions.

**FIGURE 1:**
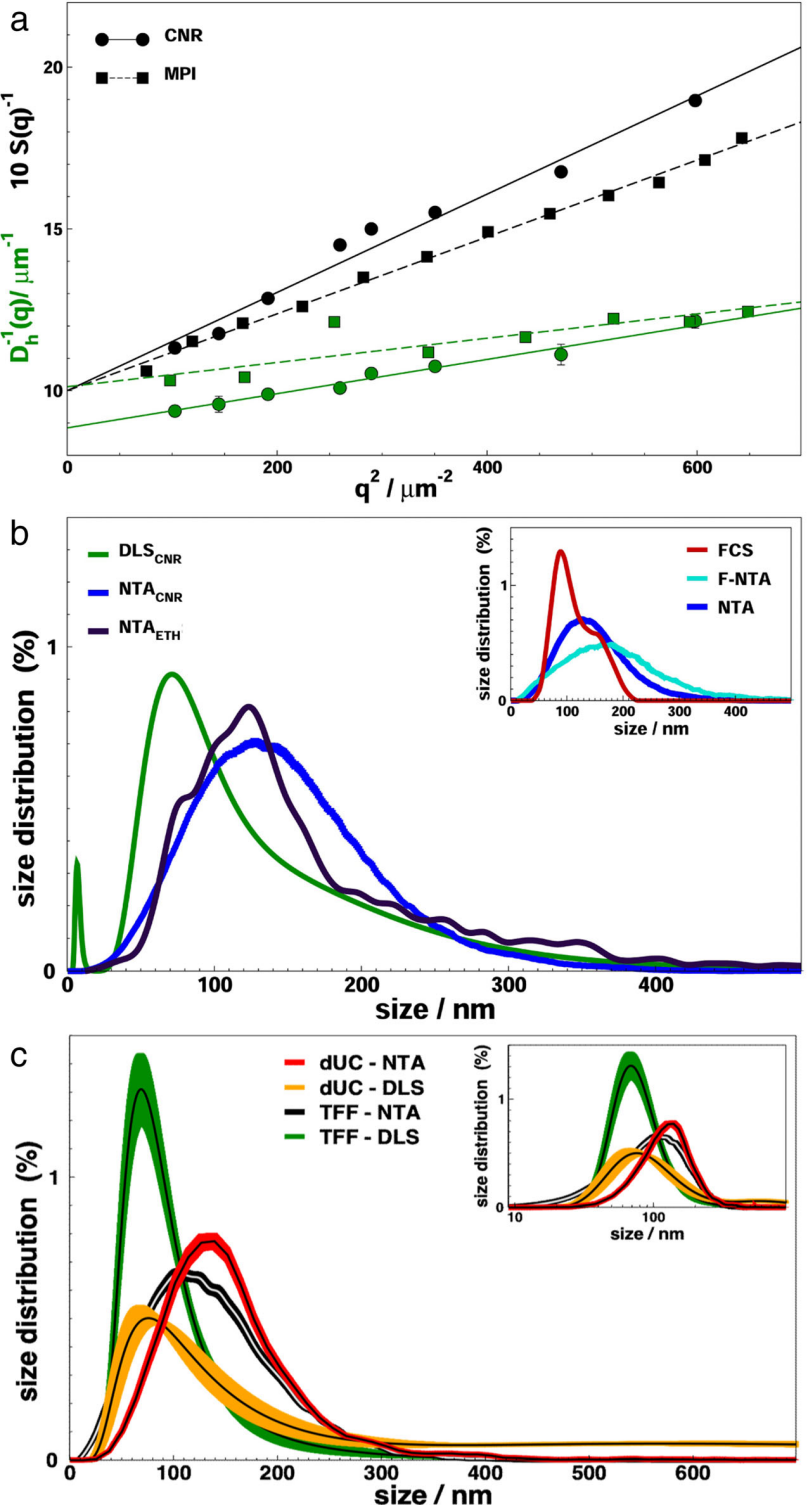
Size and size distribution of nanoalgosomes isolated by TFF. (**a**) Multi Angle Light Scattering (MALS) experiments. Form factor S(q), black, and apparent hydrodynamic diameter D_h_(q), green, as a function of the square of the scattering vector q^2^. Two experiments are reported on the same samples, performed at CNR (CNR, circles, solid curves) and MPIP (MPI, squares, dashed curves). The continuous curve represents a linear regression to data, as described in the text. The parameters D_h0_, derived from the intercept in the fit of D_h_(q)^−1^, are the z‐averaged hydrodynamic diameters; the parameters D_g_, derived from the slope in the fit of S(q)^−1^, are the double of the average radius of gyration. (**b**) Nanoalgosome size distribution by Dynamic Light Scattering (DLS), green curve, and Nanoparticle Tracking Analysis (NTA), performed at CNR, blue curve, and ETH, black curve. **Inset**: Size distribution of nanoalgosomes stained with Di‐8‐ANEPPS measured by fluorescence NTA (F‐NTA), cyan curve, and Fluorescence Correlation Spectroscopy (FCS), red curve. NTA analysis is also shown (blue). (**c**) Size distribution of nanoalgosomes isolated by TFF and dUC and analyses by DLS and NTA. The bold solid lines represent the distributions errors, that are calculated either on 5 replica of the same sample or on 3 different preparations of the same sample for NTA or DLS, respectively. Inset: Size distributions of the main panel displayed in log scale

***Multi‐angle dynamic light scattering (DLS)*** . Simultaneous static and dynamic light scattering measurements were performed at multiple scattering angles on TFF‐isolated nanoalgosomes to derive the average diameter *D_g_* and the z‐averaged hydrodynamic diameters *D_h0_*, as shown in Figure 1a. In order to validate the experimental outcomes, the experiments were repeated at two different laboratories of our consortium, by using a slightly different procedure. The average values obtained after sample filtration at MPIP (*D_g_  =  120 nm*, *D_h0_  =  100 nm*) are slightly lower than those obtained after sample centrifugation at CNR (*D_g_  =  135 nm*, *D_h0_  =* *113 nm*), since in the first case a more effective depletion of the EV population with larger size may be expected. In both cases, the ratio *D_g_/D_h0_* is about 1.2. While for monodisperse colloidal particles a value larger than 1 indicates a non‐spherical shape, for the heterogeneous nanoalgosome mixture this value is likely due to the sample polydispersity and to the asymmetric shape of its size distribution.

To account also for smaller size objects such as proteins, which were sporadically observable in the sample, from the DLS measurements we derived the complete distribution of hydrodynamic diameters *P(D_h_)* (Figure 1b). The distribution of the nanoalgosome samples are typically peaked at *D_mode_ = 70 nm* and have a tail at larger sizes; nanoalgosomes have the corresponding average size of *D_avg_ = 135 nm*. We note that the latter moment of the distribution is not equivalent to the z‐averaged size, obtained *e.g*. by cumulant method, which is a harmonic average of hydrodynamic diameters and therefore attains a lower value (*D_ho_ = 95 nm*, Figure 1b).

***Nanoparticle tracking analysis (NTA)*** . The size distribution of diluted sEV samples was measured by nanoparticle tracking which was also used to measure the particle concentration (Figure 1b). Two NTA experiments are reported on the same samples, made by using two different equipment (namely NanoSight NS300 at CNR and ZetaView at ETH). As NTA is typically more accurate on larger size particles, we observed a distribution peaked at *D_mode_ = 125 nm*, with a slightly higher average size.

***Comparison of size distribution of nanoalgosomes isolated by dUC and TFF*** . We next applied the techniques described above to compare the size distribution of dUC‐ and TFF‐isolated *Tetraselmis chuii*‐sEVs. The NTA and DLS analyses showed that both separation methods produce sEVs with comparable size distributions (Figure 1c). As a minor difference, one notes that TFF‐isolated sEVs have a slightly sharper size range with respect to dUC‐isolated sEVs. Also, a residual population of larger vesicles is more often observed in dUC‐isolated sEVs.

***Fluorescence nanoparticle tracking analysis (F‐NTA)*** . To confirm the presence of lipid membranes in the isolated nanoalgosomes, we applied F‐NTA to determine the size distribution of particles stained with Di‐8‐ANEPPS, an EV specific fluorescent dye (inset of Figure 1b). Both F‐NTA and standard (scattering mode) NTA give a largely overlapping distribution and also a comparable particle number (**Supporting Figure S3**). In addition to validate the presence of nanoalgosomes with lipid membranes, this analysis shows the absence of large number of non‐vesicle contaminants, such as lipoproteins and protein aggregates.

***Fluorescence Correlation Spectroscopy (FCS)*** . To further confirm this result, we applied the same fluorescence staining used for F‐NTA experiments to perform FCS measurements and determine the distribution of the hydrodynamic radii of fluorescent labelled species (inset of Figure 1b). It is worth noting that FCS is more accurate in detecting smaller particles with respect to F‐NTA. The analysis further confirmed the presence of lipid membranes in the isolated nanoalgosomes.

***Size distribution of nanoalgosomes by different techniques: conclusion*** . Overall, the different techniques and analyses described above provided consistent size distributions, with slight differences depending on the weight given to large and small particles and to the sampling method (**Supporting Figure S4**). With respect to NTA, DLS has no limit in the detection of diffusing vesicles also smaller than 70 nm. On the other hand, it measures a size distribution that is weighted on the square of particle mass. Thus, while the z‐averaged hydrodynamic diameter is a robust experimental parameter (multi angle DLS cumulant analysis, Figure 1a), one can obtain a more reliable esteem of the size of greatest number of particles by referring to the mode of the distribution (DLS analytic fit, Figure 1b). NTA is able to track single particles in the sample and, therefore, by definition it measures a number size distribution (NTA, Figure 1b). However, its capability for sampling small particles is affected by the detection sensibility and the tracking speed, as well as by lower light scattering of small particles, which is covered by the signal of larger particles. This determines a closer analogy between NTA distribution and the weight average distribution observed in DLS experiments. A further drawback of NTA is due to its intrinsic limitation in the lower particle range; a limitation that can be overcome by using fluorescent dyes (F‐NTA, inset of Figure 1b). When we deal with a broad size distribution, as in the present case and in the case of EVs in general, NTA represents a routinely tool to identify the average size of a broad population. Indeed, the peak observed by NTA typically mirrors the average values measured by DLS. As a second step, one can perform a more detailed DLS analysis, as we highlight in the present work, to pin point the sEV size that appears most frequently in the distribution (DLS mode).

### 3.4 Nanoalgosome identity: morphology

Since morphology is another important property of EVs, after characterizing the size distribution of our nanoalgosomes we applied different microscopy techniques to define their morphology and shape.

**FIGURE 2:**
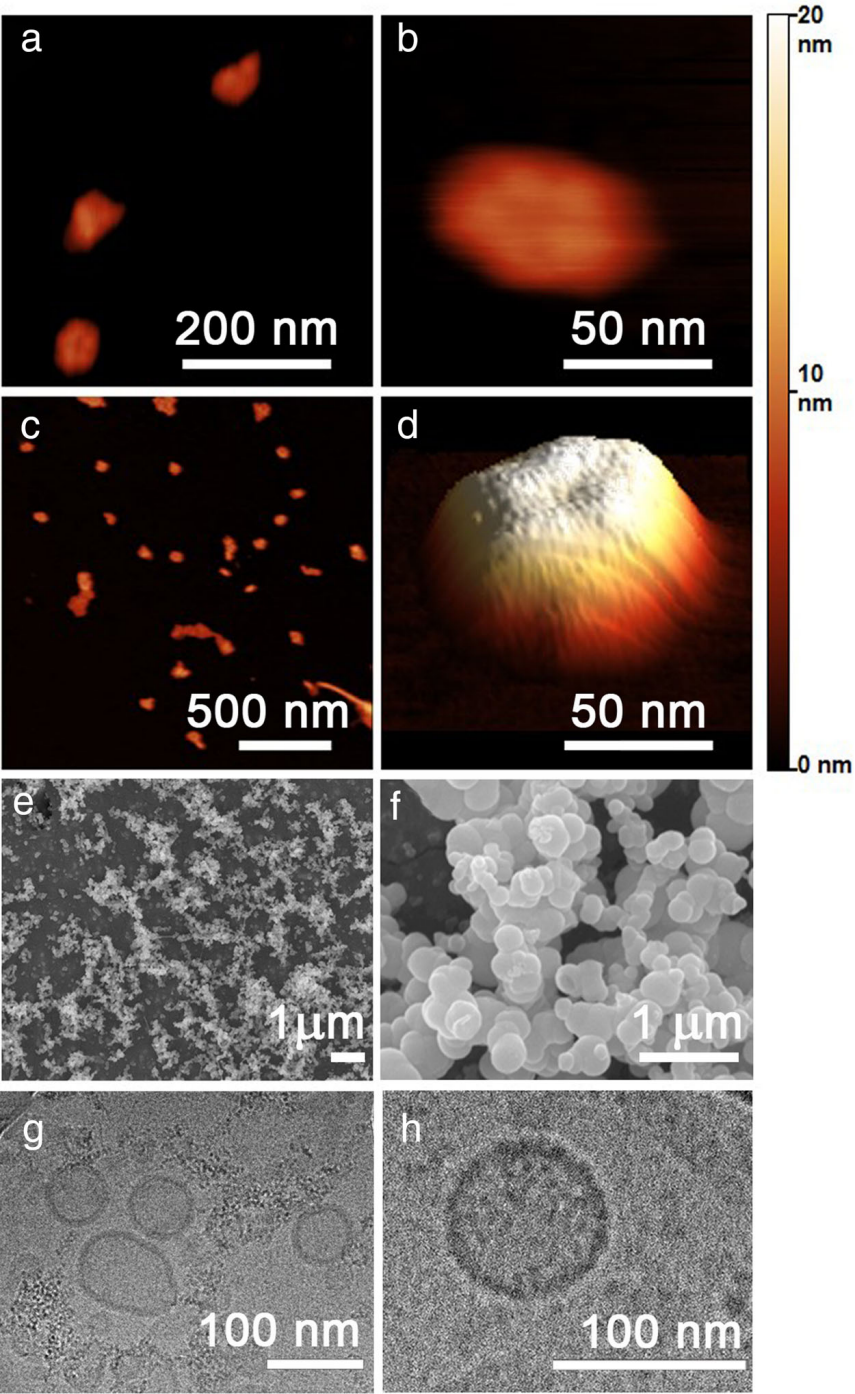
Morphology of *Tetraselmis chuii* nanoalgosomes isolated by TFF. (**a)** and **(b)** Zoom‐up tapping mode AFM images in air of selected nanoalgosomes. **(c)** Wide‐field tapping mode AFM images in air showing several sEVs. The coloured scale on the right indicates the height for all AFM images. **(d)** 3D reconstruction of a single nanoalgosomes from panel **(b)**. **(e)** and **(f)** SEM and **(g)** and **(h)** cryo‐TEM images. Representative images are presented of three independent experiments (n = 3)

***Atomic force microscopy (AFM)*** . Figures 2a‐d show AFM images of nanoalgosomes at different magnifications allowing to observe the details of single particles and the heterogeneity of a complete sEV sample. The close‐up image in Figure 2b and its 3D reconstruction in Figure 2d highlight a small dip in the middle of sEVs. This dip is caused by the strength of the AFM cantilever, which actually pushes the sEV surface causing the curvature of the membrane and of the whole particle since the edges cannot be adequately squeezed. To verify this hypothesis, we repeated the measurements with a softer cantilever and no depression was indeed observed. This observation supports the potential of AFM to study the structural and mechanical properties of EVs (Paolini et al., 2020; Ridolfi et al., 2020).

***Scanning electron microscopy (SEM)*** . SEM images of nanoalgosomes isolated from *Tetraselmis chuii* show globular particles, heterogeneous in size and shape, sometimes organized in clusters. Images of sEVs of isolates obtained by TFF are shown in Figure 2e‐f, while additional images of dUC‐ and TFF‐isolated sEVs are given in **Supporting Figures S5‐S6**.

***Cryo‐Transmission Electron Microscopy (cryo‐TEM)*** . In cryo‐TEM imaging, the nanoalgosome samples are directly applied onto an EM grid, vitrified and visualized, therefore allowing us for characterization near their native state, avoiding dehydration, chemical fixation, and/or staining which can alter the sample (as in SEM). The cryo‐TEM imaging revealed that the nanoalgosomes are spherical core‐shell nanoparticles, possessing a lipid bilayer structure, which is expected for these vesicles (Figure 2e‐f). Smaller amorphous structures, which are currently under investigation, are barely observable in the background.

### 3.5 Nanoalgosomes identity: protein markers and density

After determining size distribution, morphology and shape, following the VES4US‐MISEV guidelines, we evaluated the biochemical features of nanoalgosomes purified from *Tetraselmis chuii* culture.

**FIGURE 3:**
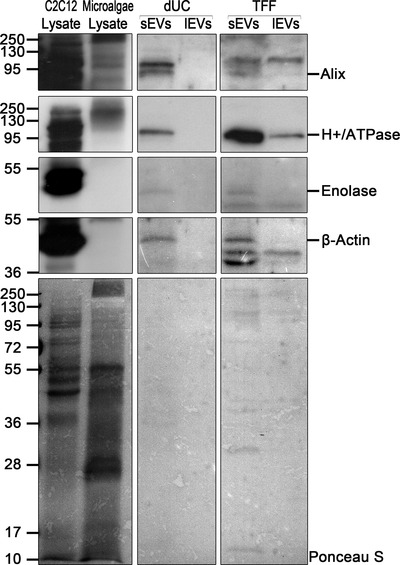
Representative immunoblot analyses of Alix, H+/ATPase, Enolase and β‐actin in *Tetraselmis chuii* cell lysates (Microalgae lysate), sEVs and lEVs isolated by dUC and TFF from *Tetraselmis chuii* cultures; a mammalian cell line is used as positive control (C2C12 lysate). [C2C12 (10 μg) and microalgae (20 μg) lysates, dUC‐isolated and TFF‐isolated sEV (13 μg) and lEV (equal volumes) samples were loaded on gels. Alix, H+/ATPase, Enolase, and β‐actin are enriched in the sEV samples. Three independent experiments (n = 3) were performed. Ponceau red staining is shown as loading control (bottom panel); lower exposures of lysate immunoblots are shown to indicate the specific bands

***Vesicle protein biomarkers*** . Selected biomarkers enriched in the EV fraction and putatively conserved during evolution were evaluated by immunoblot analyses. First, we checked for their cross‐reactivity against the relative microalgal orthologs, then, we compared their expression in the lysates as well as in the sEVs and lEVs fractions obtained by *Tetraselmis chuii*. Target protein biomarkers, chosen according with MISEV guidelines, were Alix, Enolase, HSP70, and β‐actin. This analysis was crucial to discriminate EVs from other contaminants. Immunoblot results showed the enrichment of three target proteins (i.e., Alix, Enolase, and β‐actin) in the sEV samples compared to the lysates and lEVs (Figure 3).

***Plasma membrane H+/ATPase as a biomarker of nanoalgosomes*** . The plasma membrane H+/ATPase is a transmembrane protein of about 100 kDa, which extrudes protons to generate electrochemical proton gradients, using ATP energy. The generation of this gradient is critical in providing energy for secondary active transport through the plasma membrane (Stevens & Forgac, 1997). The plasma membrane H+/ATPase plays a key role in plant physiology, in normal growth conditions and under abiotic stresses (Zhang et al., 2019). It is present also in several intracellular compartments of mammalian cells, including clathrin‐coated vesicles, endosomes, lysosomes, Golgi‐derived vesicles, chromaffin granules, synaptic vesicles, and multivesicular bodies (Han et al., 2017; Moriyama et al., 1992). It is of note that the high concentrations of solutes in secretory organelles such as chromaffin granules, synaptic vesicles, and microvesicles is allowed by specific transporters that are coupled to the proton gradient or to the membrane potential generated by the H+/ATPase. In this context, the presence of H+/ATPase was reported in human prostasomes and its expression alteration in urinary exosomes (Pathare et al., 2018). Most studies regarding H+/ATPase have been performed in plants and fungi and, due to the lack of genetic information, much less is known about plasma membrane H+/ATPase in microalgae (Pertl‐Obermeyer et al., 2018).

**FIGURE 4:**
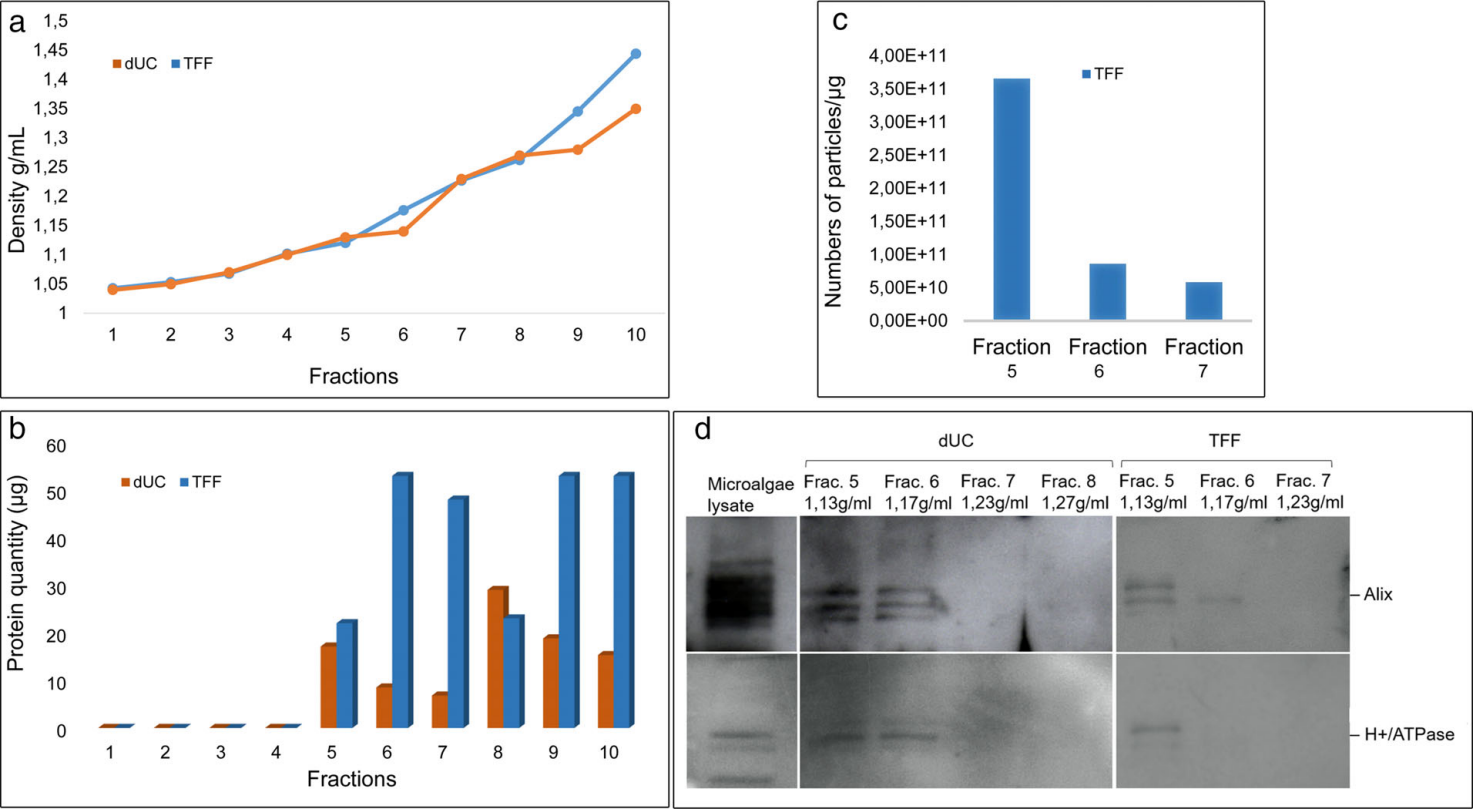
Iodixanol gradient to determine the nanoalgosome density. **(a)** The density of the ten fractions measured in the gradient ultracentrifugation (gUC) of TFF and dUC samples. **(b)** The quantity of protein measured in each gUC fraction. **(c)** The ratio of numbers of particles (determined by NTA) relative to μg of proteins measured in 5‐6‐7 gUC fractions of TFF samples. **(d)** Representative immunoblot analyses of nanoalgosomes isolated by dUC and TFF, and loaded on iodixanol density gradient. 20 μg of microalgae lysate and whole fractions were loaded on gel. Fraction 5 (density 1.13 g/ml) of dUC and TFF separated nanoalgosomes and at a less extent the fraction 6 (density 1.2 g/ml) are positive for EV specific biomarkers (Alix and H+/ATPase). Two independent biological replicas (n = 2) were performed

To find a possible biomarker of nanoalgosomes, we evaluated the presence of this highly conserved plasma membrane H+/ATPase in nanoalgosome samples. For this purpose, here, we use H+/ATPase polyclonal antibody with high reactivity and specificity for microalgae. Figure 3 shows the presence of H+/ATPase in nanoalgosome samples, isolated by TFF from two independent sEV preparations from *Tetraselmis chuii* (Figure 3). To support the specificity of H+/ATPase for sEVs, we demonstrated the presence of this protein marker in *Tetraselmis chuii* sEV fraction isolated by dUC and TFF and separated by iodixanol gradient (Figure 4d).

***Determination of nanoalgosome density*** . Density gradient ultracentrifugation in iodixanol was used to determine the nanoalgosome density and to purify and further separate sEVs isolated either by dUC or TFF. After 24 h of centrifugation, a light coloured band was observed in the tube with dUC sample but none was observed in the tube with the TFF sample. Ten fractions were collected from the top of each tube to determine the density and the protein concentration (Figure 4a).

Mammalian cell‐derived sEVs (*i.e*., exosomes) are characterized by density between 1.15 – 1.19 g/ml (Théry et al., 2006). The density of the fraction containing the visible band (Fraction 5) was measured to be slightly lower (*i.e*., 1.13 g/ml) than the density of mammalian exosomes.

Fractions 1–4 corresponding to the soluble protein fractions showed very less to no protein content in both the samples (Figure 4b). Fraction 6 (partially could be overlapping with 5 and 7) has a density of 1.12 ± 0.01 g/ml (n = 3) that corresponds to the density of mammalian exosomes showed high protein content in the TFF sample. Fraction 5 (the visible layer) has a density of 1.16 ± 0.1 g/ml showed high protein content in dUC sample. Fractions 8–10 had higher densities than sEVs, and they could be nuclei, DNA, proteins, cell fragments that co‐purify with dUC and TFF separation methods.

DUC and TFF isolated sEVs separated by gUC were analysed by immunoblotting (Figure 4d). In fractions 5 and 6 where sEVs were expected based on density, the presence of nanoalgosomes could be confirmed by Alix and H+/ATPase positivity. Based on these analyses we can conclude that the nanoalgosomes have a slightly lower density than mammalian sEVs and it is 1.13 g/ml.

### 3.6 Nanoalgosome identity: topology

The topology of different components of EVs, as recently pin‐pointed by the MISEV 2018, is an important characteristic in defining EVs functionality. Here we report a first assay performed on nanoalgosomes in regard of surface functionality.

***Amino‐groups on nanoalgosome surface*** . Amino groups on the nanoalgosome surface are originating from amino acid side chains of membrane proteins. These groups can later be utilized as an anchoring point for further functionalization. The presence of functional amino groups (‐NH_2_) on the EVs surface was quantified by a fluorescamine assay (FA) in TFF‐isolated sEVs. For the FA assay, we assumed that the diameter of sEVs is 100 nm, therefore, around 15000 ± 2500 NH_2_ groups per EV, which were isolated from *Tetraselmis chuii*, were detected. This is the first step to consider a functionalization strategy by click chemistry (Tian et al., 2018). Indeed, the presence and quantification of NH_2_ groups allow the conjugation of sEVs with linkers via the NHS ester reaction, *e.g*. NHS‐PEG_4_‐DBCO. Different biomacromolecules, *e.g*. CD11c antibody for dendritic cell targeting (Gai et al., 2020) or apolipoprotein A1 for brain endothelial cells targeting (Zensi et al., 2010), can be clicked on the EVs surface after their azidation (‐N_3_ groups) via bio‐orthogonal strain‐promoted alkyne–azide cycloaddition (SPAAC) click chemistry reaction, where Cu (I) as a catalyst can be avoided.

### 3.7 Vesicle stability

***Zeta‐potential*** . Biological membranes of cells (including EV membranes) possess a negative surface charge, mainly due to the negatively charged network of glycosylated proteins intercalated within the lipid bilayer. The surface charge of EV is reflected by its zeta potential, in turn, specific populations of EVs are expected to have certain surface charges (Midekessa et al., 2020). Large differences in zeta potential values have been reported for sEVs from different body fluids, tissues or cell cultures (Beit‐Yannai et al., 2018). For these highly heterogeneous lipid‐bilayer nanovesicles, the surface potential of the EVs, which is measured by zeta potential, is a crucial parameter for the colloidal stability study. Furthermore, it is also a key indicator for characterizing the EVs’ surface before/after functionalization with various conjugated linkers (*e.g*., antibody, protein, peptide) as these change the zeta potential depending on their own net charge. It also worth to point out that the surface charge of the EVs could be changed significantly while changing its dispersion phase conditions: *e.g*. saltwater, phosphate‐buffered saline (PBS) of various phosphate ionic concentrations (0.01, 0.1, and 1 mM), with or without detergent (Tween‐20), or in the presence of different salts (10 mM NaCl, KCl, CaCl_2_ or AlCl_3_) and at different pH values (Midekessa et al., 2020). The zeta potential of nanoalgosome samples TFF‐isolated from *Tetraselmis chuii* cultures in neutral pH (7) buffer (KCl 0.1 M) was equal to ‐13 ± 12 mV, which confirms the expectation of a negative surface charge due to the presence of proteins.

***Stability at different pH and temperature*** . DLS measurements were performed upon the same TFF isolated nanoalgosome samples buffered at different pH in the range 4–9. The size distribution of nanoalgosome sample completely unchanged over the all pH range (**Supporting Figure S7**), eliciting a striking stability at acidic and basic environments, which may be important for different biomedical applications. The same stability is preserved up to 50°C.

***Stability in biological fluids*** . The stability of EVs obtained from the microalgae *Tetraselmis chuii* was assessed in human blood plasma after 1 h incubation at 37°C. DLS measurements were subjected to a multicomponent fitting procedure as described by Rausch et al. (2010) (Rausch et al., 2010), and showed that no aggregates were present and the analyzed nanoalgosomes were stable in blood plasma (**Supporting Figure S8**). This is a key prerequisite for further application in a biomedical context.

***Stability against detergents*** . Detergents are a powerful mean to solubilize and disassemble lipid structure. To determine the detergent resistance of the nanoalgosome membranes, we incubated the nanoalgosomes with different concentrations of SDS, Triton X‐100, and Nonidet P. We then checked EV integrity by measuring the size distribution by DLS, as well as the total scattering signal, in terms of Rayleigh ratio. Our experiments show that a fraction of nanoalgosomes is destroyed upon incubation, thus giving an additional assessment to the lipid nature of the nanoalgosome envelope. Interestingly, the larger fraction of EVs resisted the incubation with detergent (**Supporting Figure S9**), while liposomes, prepared as a positive control, were immediately dissolved under the same conditions. This opens novel perspective to the study of EV resistance in relation to their membrane composition.

### 3.8 Bioactivity and cellular uptake of nanoalgosomes

Finally, after characterizing several biophysical and biochemical properties, we studied the biological activity, toxicology and cellular uptake of nanoalgosomes using a series of well‐established in vitro assays.

**FIGURE 5:**
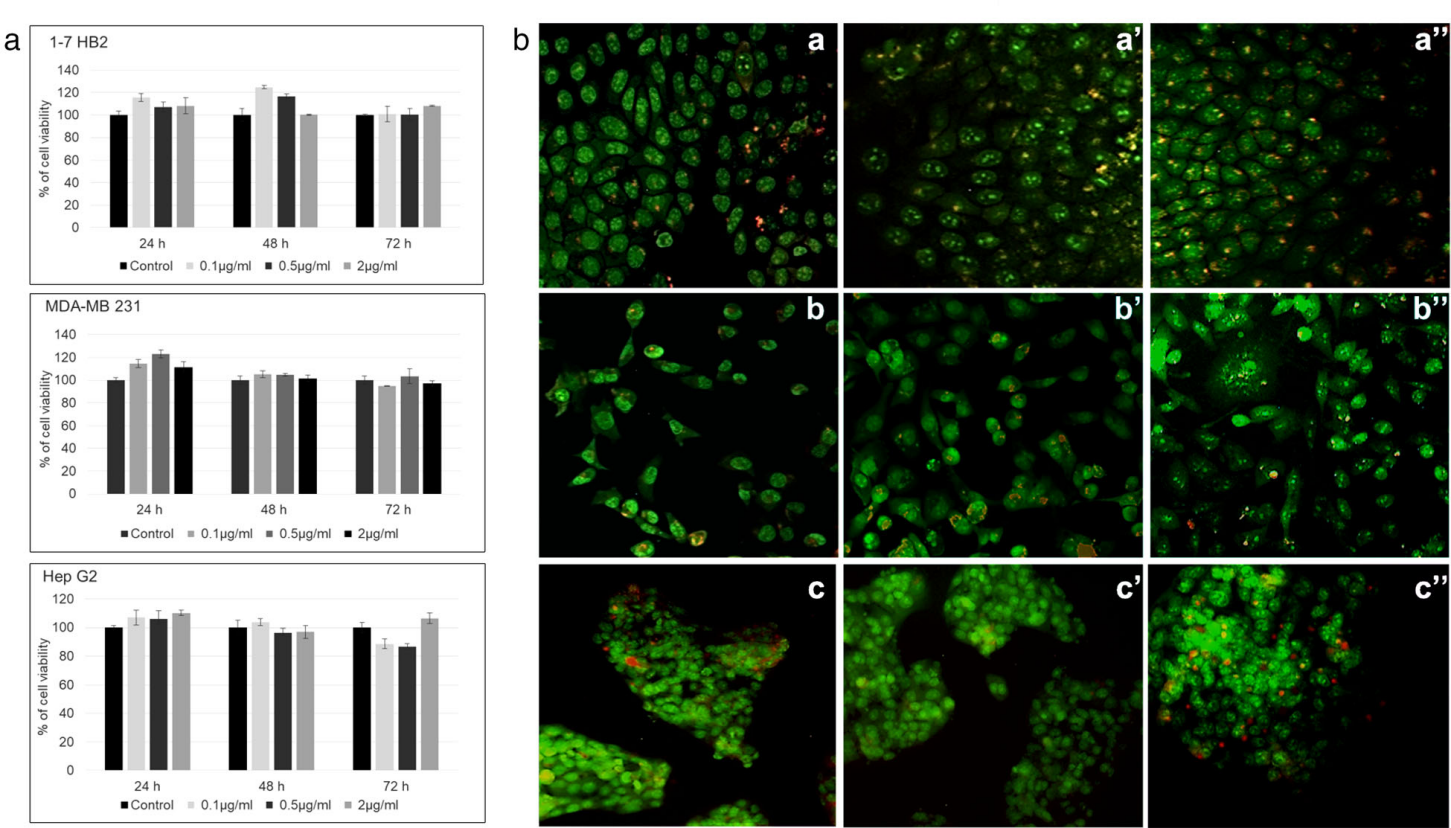
**(a)** Test of cytotoxicity activities of *Tetraselmis chuii* nanoalgosomes in: normal (1‐7 HB2), tumoral (MDA‐MB 231), and Hep G2 at different EVs concentrations (0.1‐0.5‐2 μg/ml) and for different time of incubation (24, 48 and 72 h). Cell viability is assessed by using MTS (3‐(4,5‐dimethylthiazol‐2‐yl)‐5‐(3‐carboxymethoxyphenyl)‐2‐(4‐sulfophenyl)‐2H‐tetrazolium) assays. Values were expressed as means ± SD of three independent experiments. By Student's t‐test, differences of treated cells were determined not statistically significant when compared with the control (p > 0.6). **(b)** Genotoxicity assay by Acridine Orange staining on three different cell lines: (a) untreated 1–7 HB2, (a’) 1–7 HB2 treated with 2 μg/ml *Tetraselmis chuii* sEVs for 48 h and (a’’) 1–7 HB2 treated with 2μg/ml *Tetraselmis chuii s*EVs for 72 h; (b) untreated MDA‐MB 231, (b’) MDA‐MB 231 treated with 2μg/ml *Tetraselmis chuii* sEVs for 48 h and (b’’) MDA‐MB 231 treated with 2μg/ml *Tetraselmis chuii* EVs for 72 h; (c) untreated Hep G2, (c’) Hep G2 treated with 2μg/ml *Tetraselmis chuii* EVs for 48 h and (c’’) Hep G2 treated with 2μg/ml *Tetraselmis chuii* EVs for 72 h. Representative images of three independent experiments are showed. Magnification 20X

***Toxicity assays*** . The cytotoxicity of microalgal sEVs was evaluated by the cell viability MTS (3‐(4,5‐dimethylthiazol‐2‐yl)‐5‐(3‐carboxymethoxyphenyl)‐2‐(4‐sulfophenyl)‐2H‐tetrazolium) assay. Nanoalgosomes derived from *Tetraselmis chuii* did not show significant toxicity both on the tumorigenic MDA‐MB 231 breast cancer cell line and on the non‐tumorigenic 1–7 HB2 mammary luminal epithelial cell line, over time and at different concentration (Figure 5a).

For the genotoxicity assay, we used the Acridine Orange (AO) staining. As it can be observed in Figure 5b (a‐a’’), 1–7 HB2 cells, treated or untreated, show a normal epithelial disposition and cell shape, with well‐organized nuclear structures and no signs of DNA damage (i.e., the number of damaged red nuclei were unchanged compared to control cells). Also, MDA‐MB 231 cells show uniform bright green nuclei with organized structures, similarly to the untreated cells, excluding the classical morphological changes associated with apoptotic events, also after 72 h of treatment (Figure 5b, b‐b’’), in agreement with the cell viability assay.

Subsequently, we tested the effect of *Tetraselmis chuii* nanoalgosomes on human hepatocarcinoma cell (Hep G2 cell line), as a useful model to obtain data on any EV‐effects on the metabolism of cells of hepatic origin and to assess the risk of hepatotoxicity. To this aim we analysed the in vitro hepatotoxicity by using the MTS assay (Figure 5a) and observed no significant toxic or metabolic effects induced by the microalgal sEVs on the hepatic cells, at different concentrations and over time (Figure 5a). The AO staining was used for hepato‐genotoxicity evaluations, following treatment with microalgal EVs. Figure 5b (c‐c’’) shows the AO images of Hep G2 cells incubated with *Tetraselmis chuii* nanoalgosomes, for 48 or 72 h. Also in this case, we observed no genotoxic changes after nanoalgosome treatment with respect to the control ones.

**FIGURE 6:**
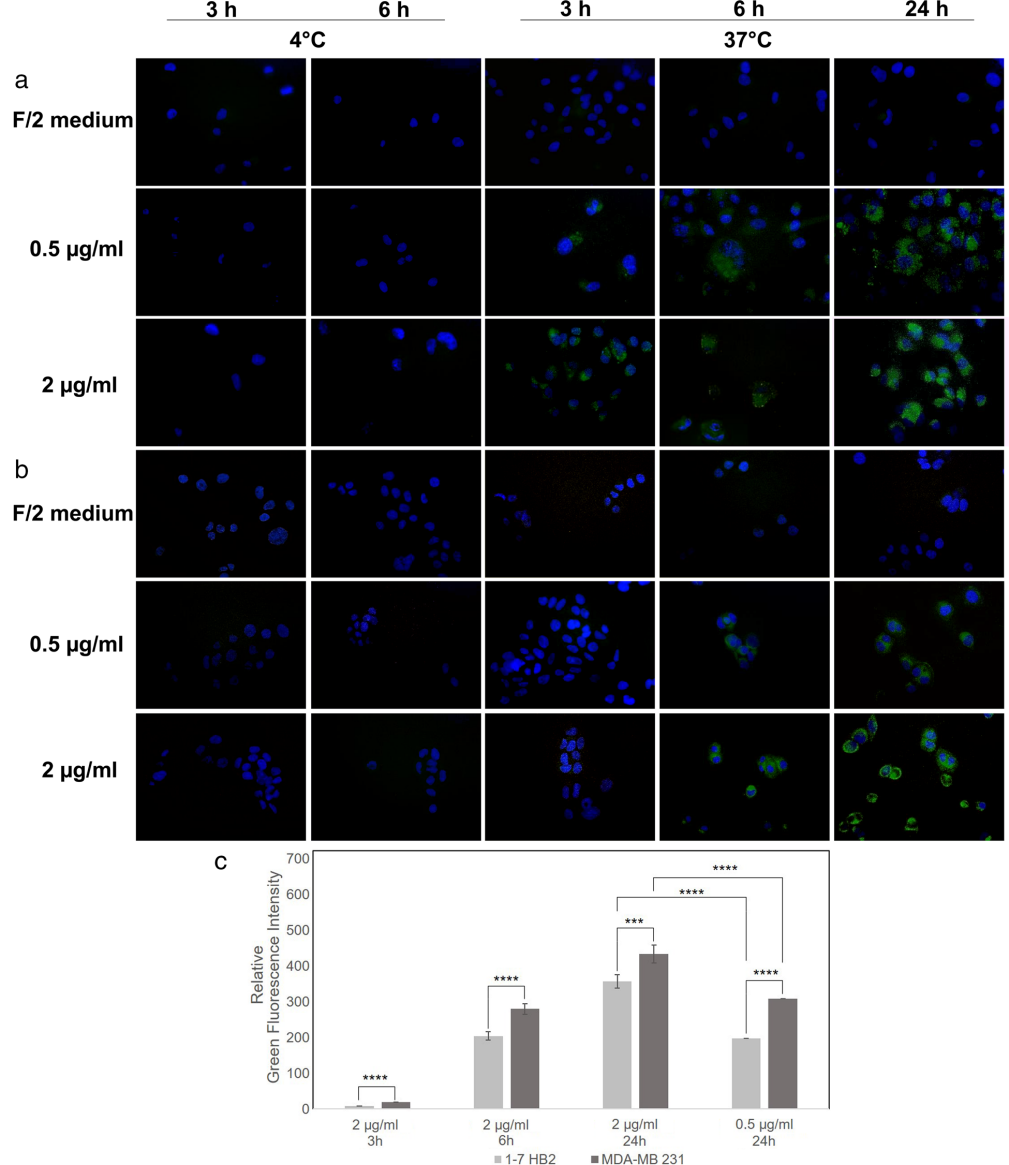
Representative fluorescence microscopy images showing the cellular uptake of Di‐8‐ANEPPS fluorescent nanoalgosomes (green) in MDA‐MB 231 **(a)** and 1–7 HB2 **(b)** cell lines (nuclei in blue), incubated with different concentrations of f‐EVs at 37°C for 3, 6 and 24 h. DUC‐isolates using not‐conditioned f/2 media and stained with DI‐8‐ANEPPS and 4°C incubation are shown as negative controls. Magnification 40X. **(c)** The relative green fluorescence intensities of MDA‐MB 231 and 1–7 HB2 cell lines incubated with 2 μg/ml f‐EVs, at 37°C for 3, 6 and 24 h, or with 0.5 μg/ml f‐EVs at 37°C for 24 h are reported as relative values against the green fluorescent intensities of cells treated with the dUC‐isolates from not‐conditioned f/2 media at 37°C for 24 h. The data are presented as means ± SD (*** *P* < 0.001, **** *P* < 0.0001)

**FIGURE 7:**
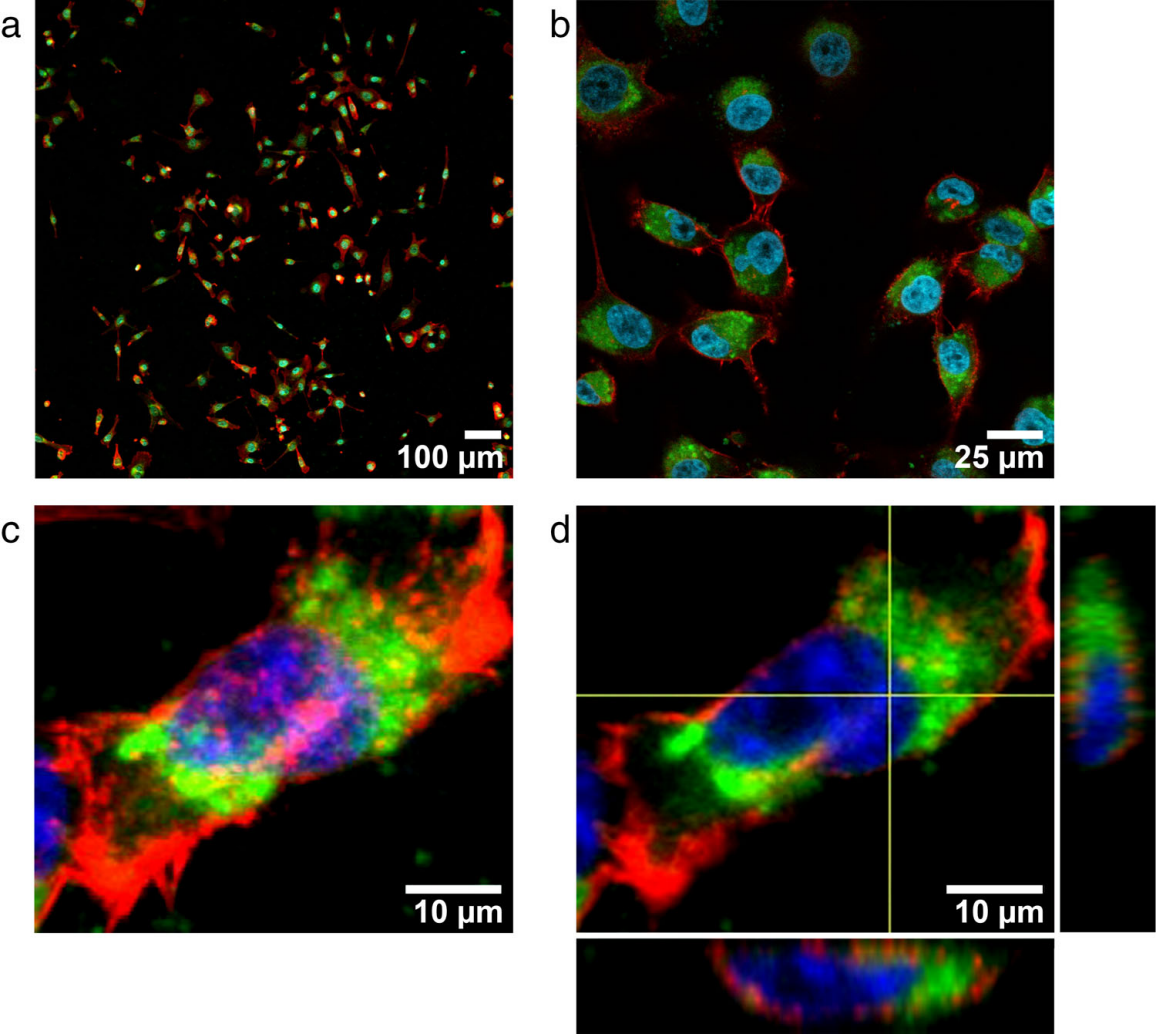
Confocal microscopy analysis of nanoalgosome internalization in MDA‐MB 231 cells (nuclei stained with DAPI in blue; F‐actin stained with Alexa Fluor 594 phalloidin in red), incubated with 2 μg/ml of Di‐8‐ANEPPS fluorescent nanoalgosomes (green) at 37°C for 24 h. (**a‐b**) Representative confocal microscopy images showing optical mid‐sections (at focal depth of 7 μm over a total scanning thickness of ∼15 μm), at 10x and 60x of magnification, respectively. (**c**) Representative confocal Z‐stack acquisition and (**d**) its orthogonal projections. Scale bars are showed. Three independent biological replicas were performed

***Cellular uptake*** . Once established that *Tetraselmis chuii* EVs are not cytotoxic, hepatotoxic or genotoxic at different concentrations and timing, we have determined the cross‐kingdom communication among the microalgal sEVs and the human cells, by testing the cellular uptake. Cells were incubated with different concentrations of Di‐8‐ANEPPS‐stained *Tetraselmis chuii*‐derived sEVs (f‐EVs) (*i.e*., 0.5 and 2 μg/ml) for different incubation times, namely 3, 6 and 24 h. To confirm that nanoalgosomes actively bypass cell membrane and that they were uptaken through an energy dependent mechanism, we incubated cells at 4°C, as negative control. As shown in Figure 6a, the f‐EVs rapidly bypass the cellular membrane in MDA‐MB 231 cells, to accumulate intracellularly after 3 h, only at the temperature of 37°C. Their distribution in the cytoplasm compartment was mostly evident after 24 h (Figure 6a,c), respect to samples incubated at 4°C in which there were no detectable fluorescence (Figure 6a, left rows). Similarly, the normal 1–7 HB2 cells have uptaken the f‐EVs, although slowly (> 6 h) and in a significant lower amount (Figure 6b‐c). In addition, we excluded aspecific green fluorescence by repeating the same studies using as a further negative control the dUC‐isolates using not‐conditioned f/2 media and stained with DI‐8‐ANEPPS. No fluorescence signal was detected from negative control images, for all the conditions analysed (Figure 6a‐b). These results are confirmed by confocal analyses that show the intracellular localization of fluorescent nanoalgosomes in MDA‐MB 231 cells (Figure 7a‐d). Thus, we could conclude that both human cell lines were able to uptake nanoalgosomes in a specific dose‐ and time‐dependent manner (Figure 6a‐c, 7a‐d).

### 3.9 Discussion

Nanoalgosomes are novel membranous biogenic nanomaterial refined for the first time from a sustainable and renewable bioresource (i.e., microalgae), which can be used as a new natural delivery system for high‐value microalgal substances (such as antioxidants, pigments, lipids and complex carbohydrates), bioactive biological molecules (e.g., proteins, miRNA, siRNA, mRNA, lncRNA, peptides) and/or synthetic drugs.

Different separation procedures (*e.g*., dUC, TFF and gUC) allowed to isolate nanoalgosomes from the *Tetraselmis chuii*‐conditioned media. As experimentally demonstrated by their sensitivity to the detergent SDS and their positivity to the membrane staining with Di‐8‐ANEPPS, we could conclude that nanoalgosomes are biogenic lipidic membranous nanovesicles. They contain one or more established EV protein markers, such as Alix and the additional protein marker, plasma membrane H+/ATPase, and can be efficiently and safely taken up by mammalian cells, confirming the cross kingdom communication potential of EVs (Gill et al., 2019). The study presented here is promising in using nanoalgosomes in therapy. Also, future in vivo, pre‐clinical and clinical testing would allow to evaluate whether nanoalgosomes are not toxic, low immunogenic, and well‐tolerated by the organism. Further, their engineering is already ongoing to confer targetability feature to specific cell or tissue.

Also, we have been able to isolate EV‐like nanoparticles from several microalgae strains. In particular, nanoalgosomes were purified and characterized from two other strains: the *Tetraselmis chuii*‐related chlorophyte *Dunaliella tertiolecta*, and the distant phytoplankton group dinoflagellate *Amphidinium sp*. (**Supporting Figure S10**). Despite the polyphyletic origin, we can conclude that the nanoalgosome production is an evolutionary conserved trait within this heterogeneous group of protistean organisms.

It is well known that EVs constitute vehicles for inter‐organisms communication. For instance, bacterial EVs perform several functions, including molecular transport, mediation of stress response, biofilm formation and the influence on hosts (Bleackley et al., 2020; Cai et al., 2018; Gill et al., 2019; Muraca et al., 2015; Soares et al., 2017; Yáñez‐Mó et al., 2015). Vesicle release has been also observed in a variety of cultured marine cyanobacteria (Biller et al., 2014). Further, field studies demonstrated that in natural environments, such as the aquatic ecosystem, bacterial‐derived extracellular vesicles are abundant, and they likely play a role in the ecology of marine microbial ecosystems (Biller et al., 2014). It has been postulated that the cyanobacteria vesicles can serve as food parcels for marine organisms and/or as a defence agent against phage attack (cellular decoys), and with implications for marine carbon cycling, mechanisms of horizontal gene transfer (Biller et al., 2014). The concentration of vesicles would vary from place to place in ocean ecosystems, reflecting the balance between species‐specific production rates, degradation rates, and consumption rates by the marine food web. In this context, future studies may help to establish the biological function of the extracellular vesicles produced by microalgae, the most abundant primary unicellular organism found in all the aquatic systems.

**FIGURE 8:**
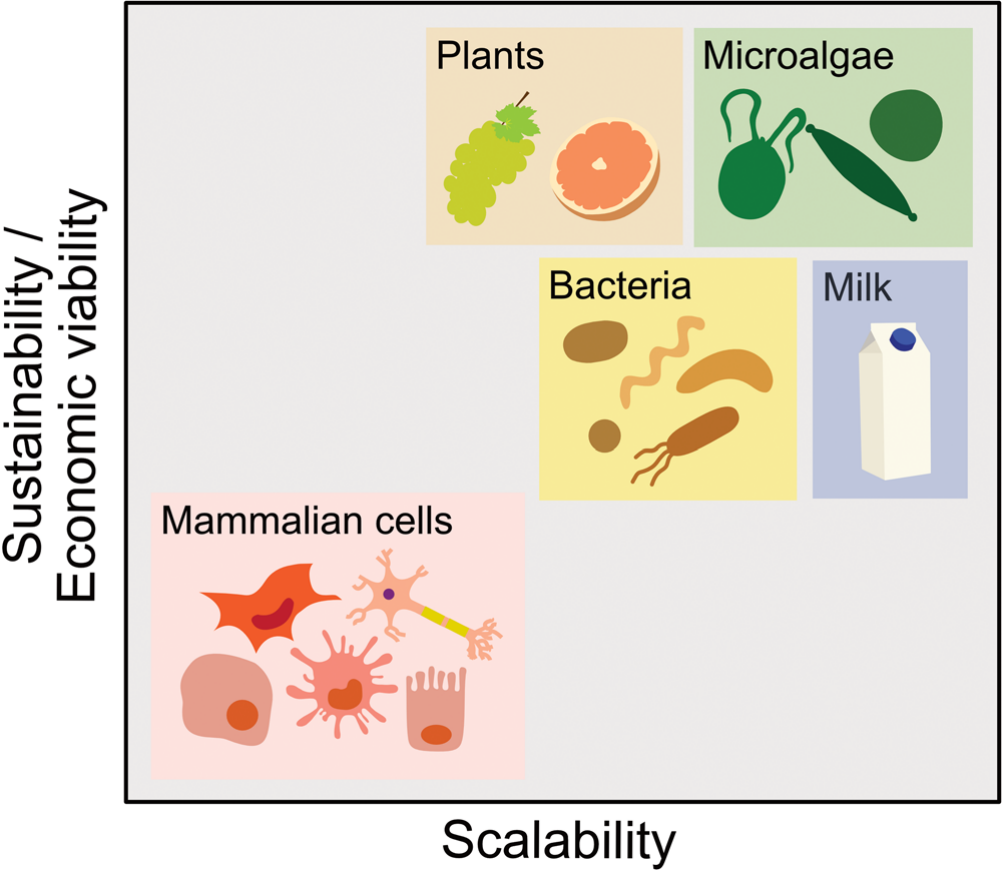
The scalability, sustainability and economical viability of nanoalgosome production compared to the most common sources of therapeutic vesicles. [The ranking represents an arbitrary evaluation based on the analysis of the nanoalgosome production in the context of current bioprocesses (Paganini et al., 2019)]

The use of microalgae as a natural source for EVs would provide a number of advantages. Indeed, the metabolic attributes of microalgae are actively researched worldwide to address strategic priorities, with a particular focus on biofuel generation, bioremediation developments and biosynthesis of high‐added value biochemicals (Blanch, 2012; Khozin‐Goldberg et al., 2016; Pulz & Gross, 2004; Sun et al., 2013; Yáñez‐Mó et al., 2015). Recent advances in microalgal biotechnology have generated much optimism for a viable industrial production of microalgae‐derived compounds such as antioxidants or omega‐3 polyunsaturated fatty acids. Nanoalgosomes presented here would offer a number of advantages compared to mammalian cell‐, plant‐, bacteria‐, and milk‐derived EVs in that microalgae cells have high growth rates, can be cultured on non‐arable land under controlled environmental conditions in large scale photobioreactors and produce nanoalgosomes with a yield comparable to other sources (Bitto & Kaparakis‐Liaskos, 2017; Gerritzen et al., 2017; Kim et al., 2015; Munagala et al., 2016; Paganini et al., 2019) (Figure 8; Pocsfalvi et al., 2018; Raimondo et al., 2015; Wang et al., 2013). In addition, the natural and sustainable origin of nanoalgosomes grants them a likely greater societal acceptance (with reduced sensitive ethical questions) as a source for formulation preparations.

## DISCLOSURE STATEMENT

The authors report no conflict of interest.

## Supporting information

Supporting information.
