# Supplementary material for: Nanoalgosomes: Introducing extracellular vesicles produced by microalgae
Source: J Extracell Vesicles. 2021 Apr 27;10(6):e12081. doi: 10.1002/jev2.12081 (PMC8077145; doi:10.1002/jev2.12081)
Supplement: Supplementary file 1 — Supporting information. [file JEV2-10-e12081-s001.zip › jev212081-sup-0001-SuppMat.pdf]

# Supporting Figures

## Nanoalgosomes: introducing extracellular vesicles produced by microalgae

Giorgia Adamo[1,a], David Fierli[2,a], Daniele P. Romancino[1,a], Sabrina Picciotto[1,a], Maria E. Barone[2,a], Anita Aranyos[2,a], Darja Božič[3,a], Svenja Morsbach[4,a], Samuele Raccosta[5,a], Christopher Stanly[6,a], Carolina Paganini[7,a], Meiyu Gai[4], Antonella Cusimano[1], Vincenzo Martorana[5], Rosina Noto[5], Rita Carrotta[5], Fabio Librizzi[5], Loredana Randazzo[5], Rachel Parkes[2], Umberto Capasso Palmiero[7], Estella Rao[5], Angela Paterna[5], Pamela Santonicola[6], Ales Iglič[3], Laura Corcuera[8], Annamaria Kisslinger[9], Elia Di Schiavi[6], Giovanna L. Liguori[10], Katharina Landfester[4], Veronika Kralj-Iglič[3], Paolo Arosio[7], Gabriella Pocsfalvi[6], Nicolas Touzet[2], Mauro Manno[5,b,#] and Antonella Bongiovanni[1,b,#]

[1]Institute for Research and Biomedical Innovation (IRIB) - National Research Council of Italy (CNR), Palermo, Italy
[2]Centre for Environmental Research Innovation and Sustainability Institute of Technology Sligo, Sligo, Ireland
[3]University of Ljubljana (UL), Ljubljana, Slovene
[4]Max Planck Institute for Polymer Research (MPIP), Mainz, Germany
[5]Institute of Biophysics (IBF) - National Research Council of Italy (CNR), Palermo, Italy
[6]Institute of Biosciences and BioResources (IBBR) - National Research Council of Italy (CNR), Naples, Italy
[7]Department of Chemistry and Applied Biosciences, ETH Zurich, Zurich, Switzerland
[8]Zabala Innovation Consulting, Pamplona, Spain
[9]Institute of Experimental Endocrinology and Oncology (IEOS) - National Research Council of Italy (CNR), Naples, Italy
[10]Institute of Genetics and Biophysics (IGB) - National Research Council of Italy (CNR), Naples, Italy

[a]These first Authors contributed equally to this work
[b]These last Authors contributed equally to this work

#Corresponding Authors: antonella.bongiovanni@cnr.it; https://orcid.org/0000-0002-0307-4043; mauro.manno@cnr.it; https://orcid.org/0000-0001-9843-0428

*All the listed Authors are members of the VES4US consortium (H2020 grant agreement #801338)

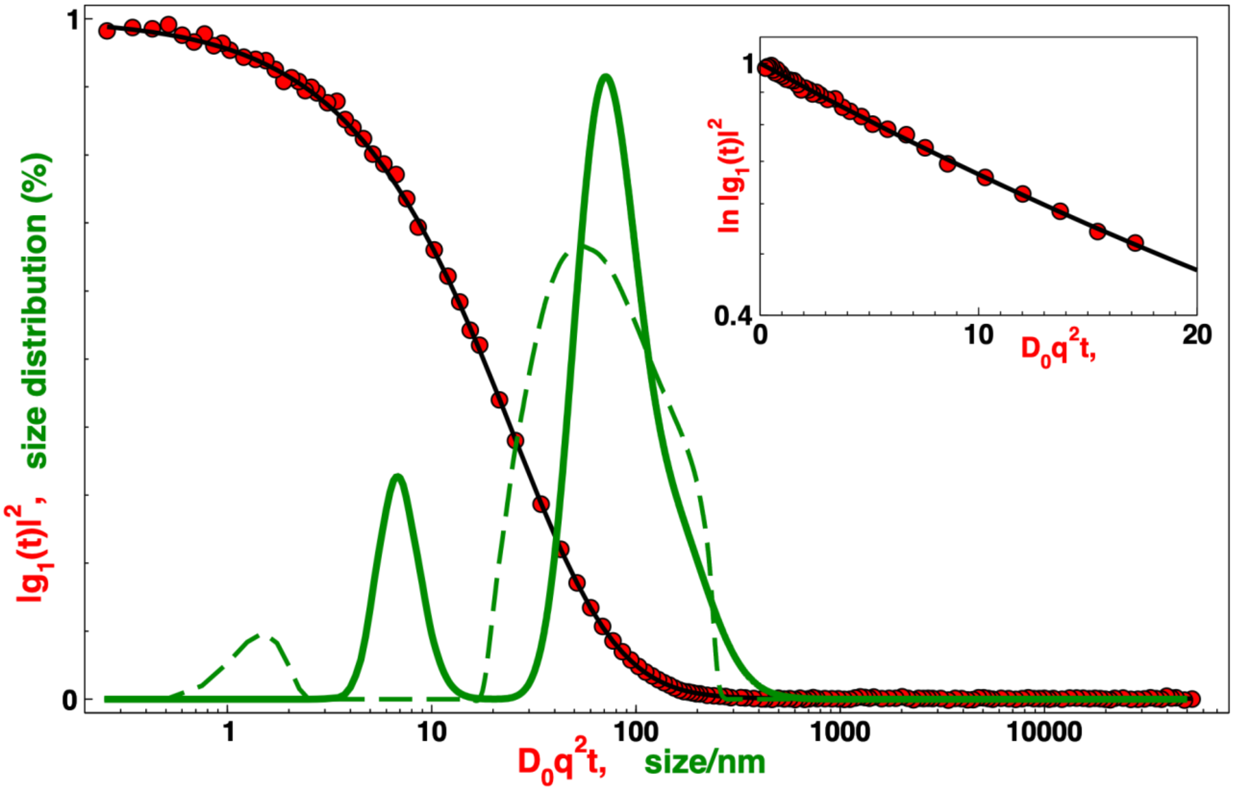


**Figure S1. Dynamic Light Scattering (DLS) data analysis.**

Squared modulus of the scattering electric field correlation function at 90°, $g_1(t)$, for the same sample of Figure 1 (at CNR), red circles; the solid line is an analytic fit to data; the time axis is made consistent with the size axis by multiplying the correlation time *t* by $(D_0q^2)$, where $D_0$ is the diffusion coefficient of *1 nm* particle. The size distribution obtained by an analytic fit, as described in the text, is shown as a solid green curve; the size distribution obtained by a regularization fit based on CONTIN algorithm is shown as a dashed green curve. Inset: cumulant analysis (solid curve) of the data from the main panel (red circles); the polydispersity index is 0.3. A representative measurement and analysis of *Tetraselmis chuii* nanoalgosomes (n=20).

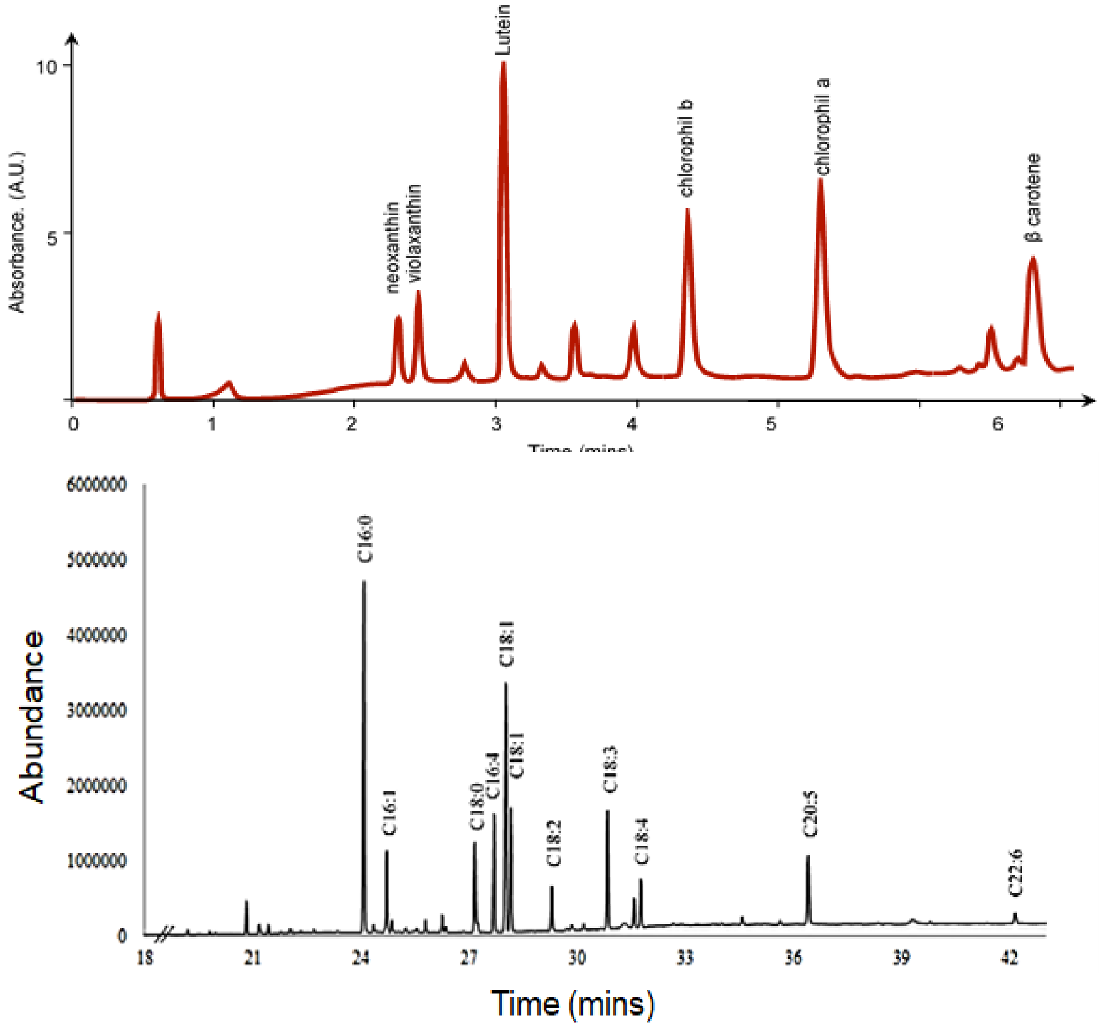


**Figure S2. *Tetraselmis chuii* pigment and FAME analysis.**

**Upper panel**. HPLC-UV-DAD chromatogram obtained for an extract of the clorophyte *Tetraselmis chuii* indicating the main pigments detected. Several pigments typical of chlorophyte species were identified, including Neoxanthin, Violaxanthin, Lutein, Chlorophyll a, Chlorophyll b, and β carotene.

**Lower panel**. GC/MS chromatogram obtained for an extract of the clorophyte *Tetraselmis chuii* indicating the fatty acids detected. The fatty acid methyl ester (FAME) signature was analysed by GC-MS, and the following fatty acids were identified: C16, C16:1, C16:4, C18, C18:1, C18:2, C18:3, C18:4, and the high-value long chain PUFAs EPA (C20:5).

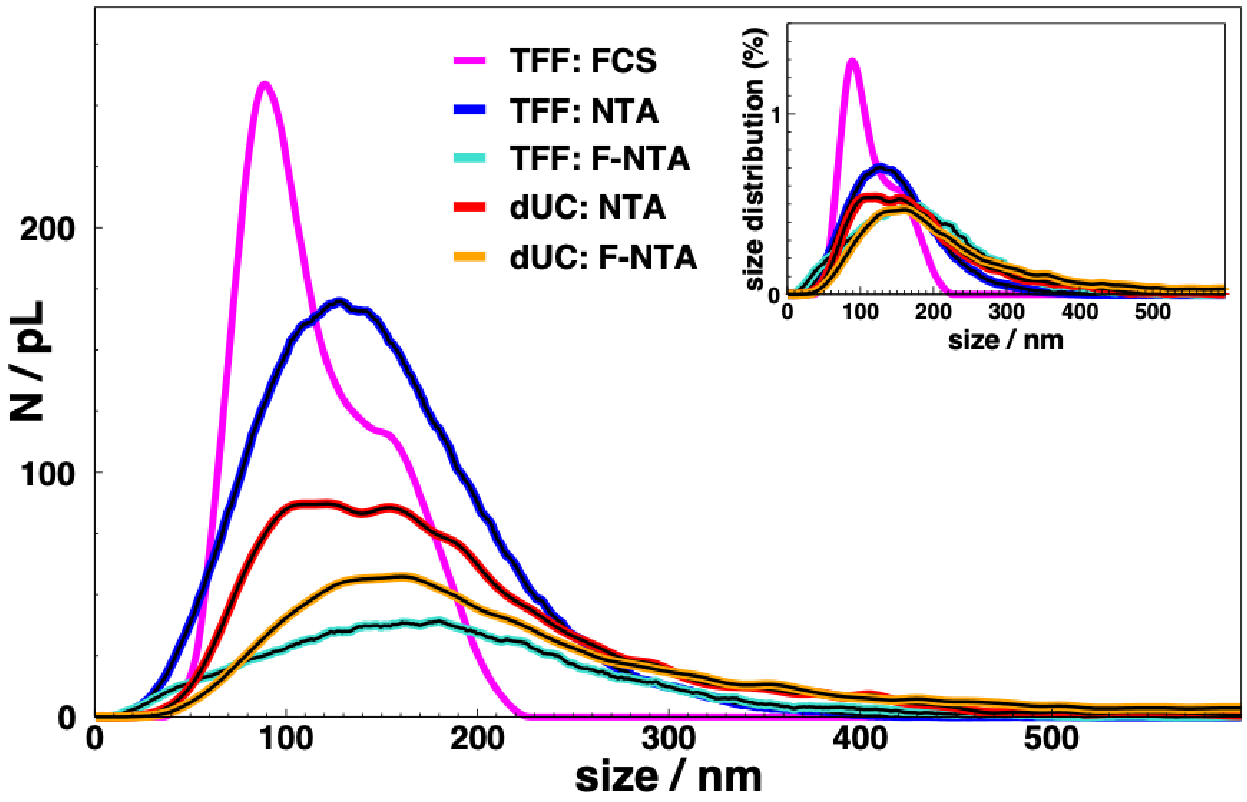


**Figure S3. Size distributions of nanoalgosomes by fluorescence based techniques.** Size distributions of nanoalgosomes isolated by dUC and stained with Di-8-ANEPPS; the sample is measured by standard NTA (blue curve, TFF; red curve, dUC), F-NTA (cyan curve, TFF, orange curve, dUC) and FCS (magenta curve, TFF). The distribution are normalized to the total particle number. Inset: The same distribution as in the main panel, normalized to 100. A representative measurements and analyses of dUC- (n=2) and TFF-isolated (n=2) *Tetraselmis chuii* nanoalgosomes.

A

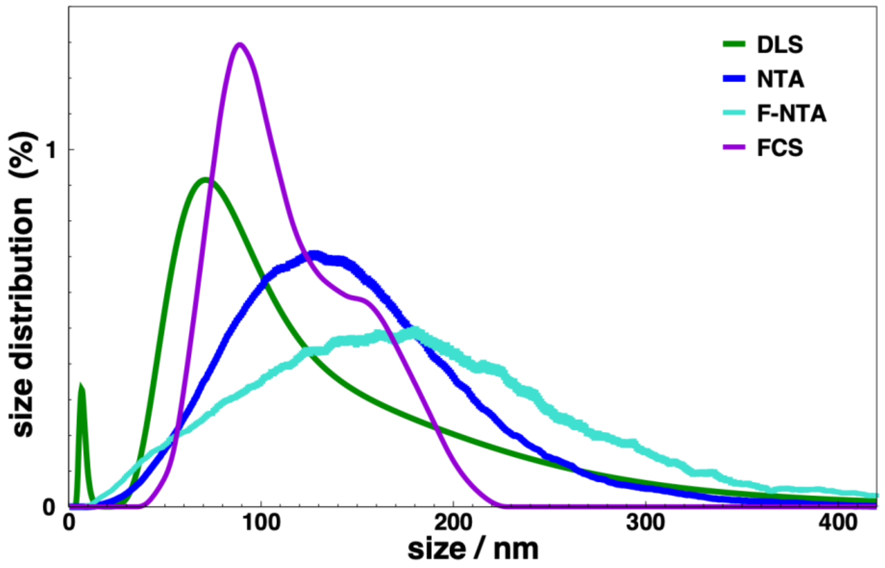


B

| Technique | | moda | average | std. deviation |
|---|---|---|---|---|
| Multi Angle DLS | *Contin @MPI* | $R_h$ = 100 ± 10 nm, $R_g$ = 120 ± 10 nm | | |
| | *Cumulants* | $R_h$ = 115 ± 10 nm, $R_g$ = 135 ± 10 nm | | |
| NTA | | 125 ± 5 nm | 150 ± 10 nm | 70 ± 10 nm |
| FCS | | 90 ± 5 nm | 115 ± 10 nm | 40 ± 10 nm |
| DLS | | 70 ± 5 nm | 135 ± 10 nm | 110 ± 10 nm |

**Figure S4.** (**A)** Size distribution of nanoalgosomes isolated by TFF obtained with different techniques (i.e., NTA, DLS, FCS, F-NTA). **(B)** Table with significant size distribution parameters. In Multi Angle DLS and FCS the parameters are related to a single experiment (Figure1, panel A); in DLS and NTA the parameters are the average over two sets of measurements on TFF isolated nanoalgosomes (n=14).

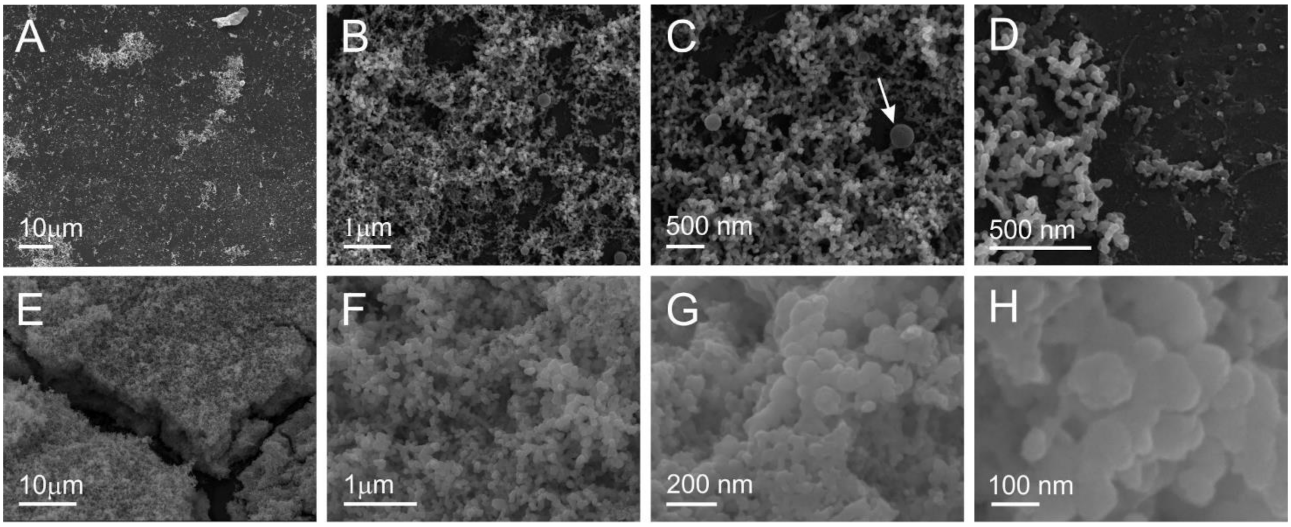


**Figure S5.** SEM images of *Tetraselmis chuii* nanoalgosomes obtained by ultracentrifugation. Series **A-D** and **E-H** represent images of the sEV-deposited landscape with increasing magnification, obtained for n=2 independent technical replicas, respectively.

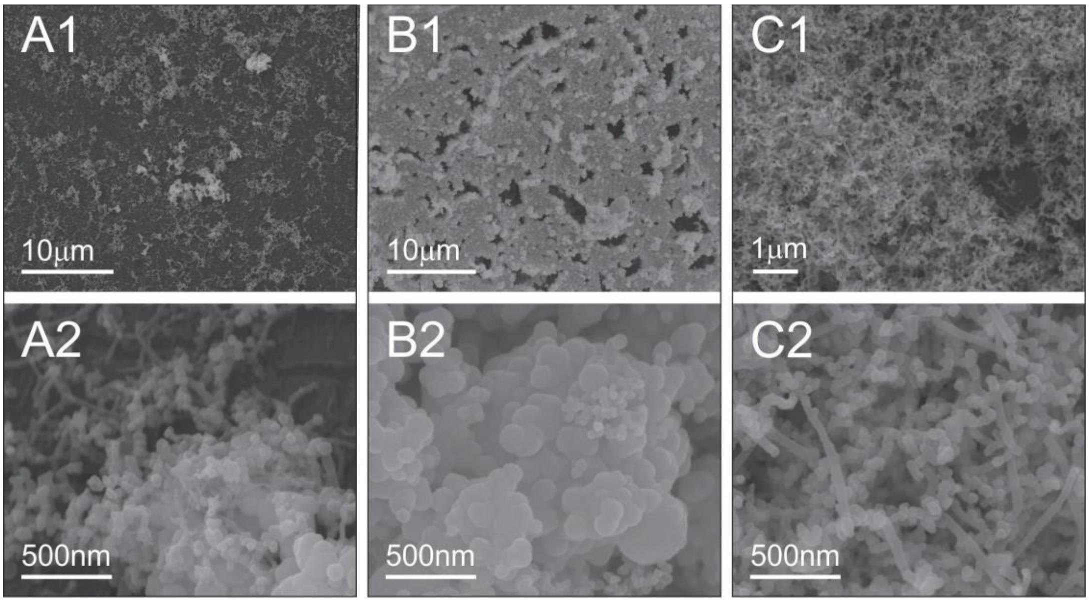


**Figure S6.** SEM images of the sEVs obtained by TFF, starting from 7.5 L of *Tetraselmis Chuii* conditioned media. First row (marked by the number 1) shows lower magnification of isolate landscape while the second row (marked by the number 2) shows higher magnification of the same region. **A**: the sample was rich with sEVs, heterogeneous in size and shape, however, it can be estimated that the diameter of the globular particles is on the average 100 nm. **B**: deposits of larger particles of more smooth spherical shape, **C**: tubular structures of which some appear undulated; either the globular particles are formed by undulation of the tubes into the vesicles and separation of the vesicles, or the tubes are formed by assembly of the globular particles.

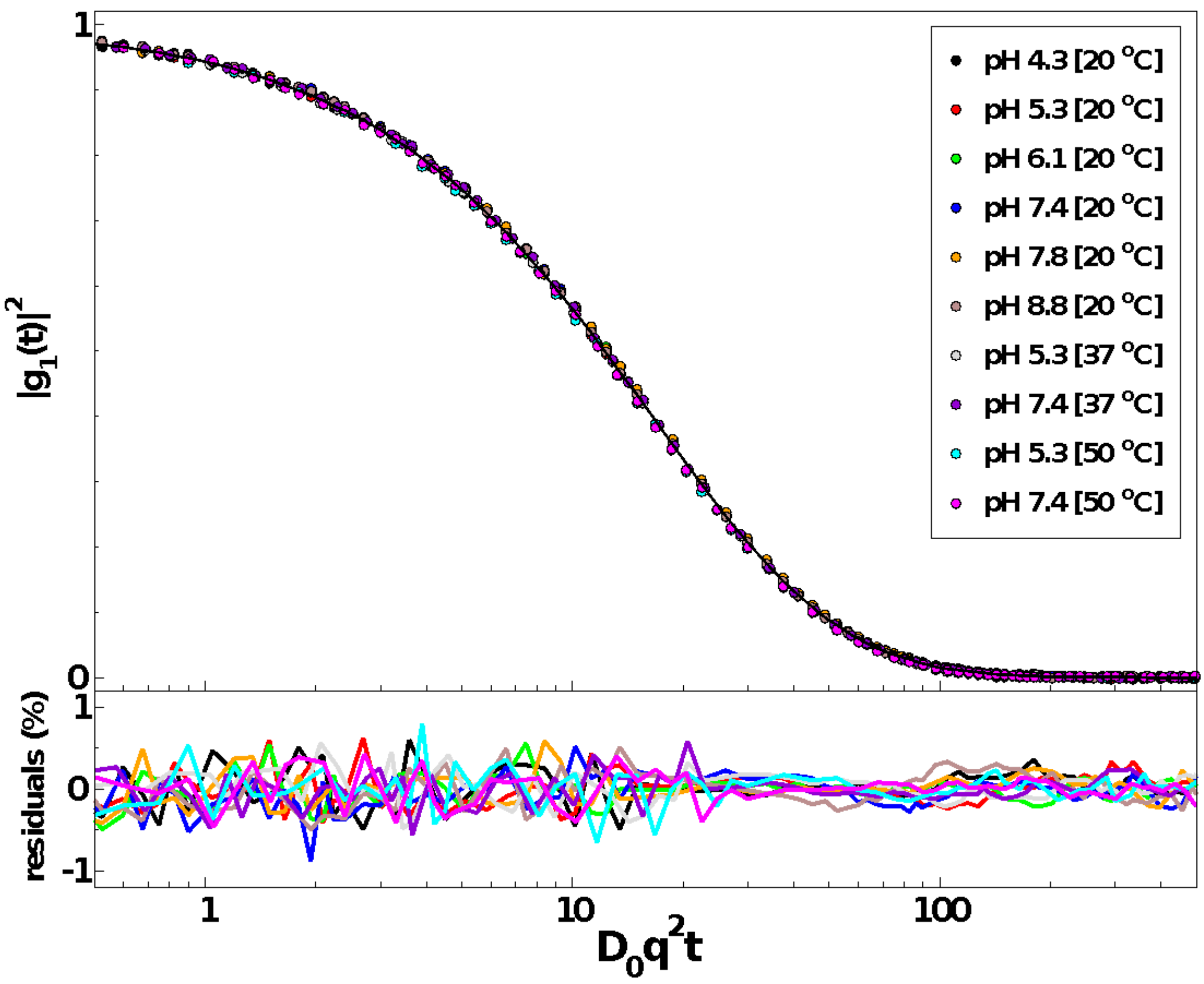


**Figure S7.** Stability of sEVs isolated from the microalgae *Tetraselmis chuii* at different pH and temperatures. Upper panel: DLS correlation functions at scattering angles of 90° of sEVs at different pH and temperatures (colored circles, as in the legend), along with a fit (solid black curve). Residual between fit and data are shown in the lower panel.

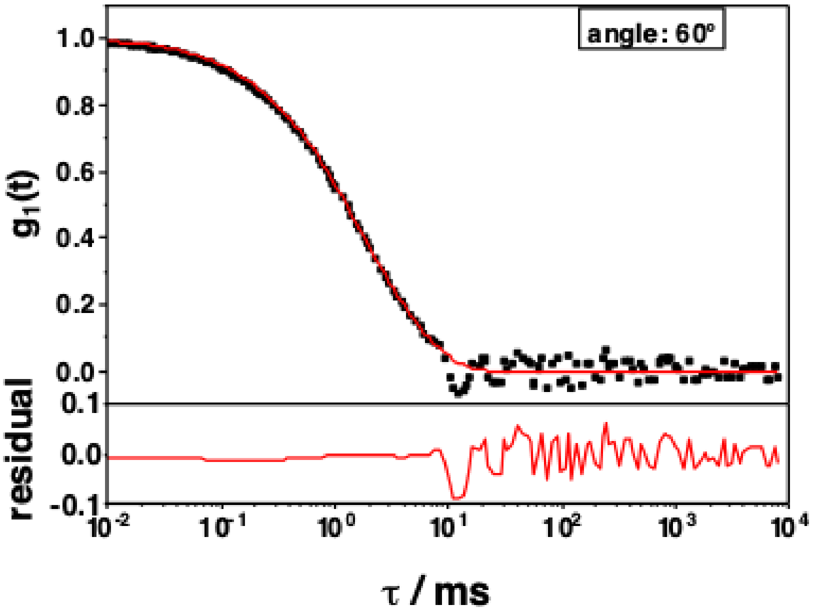


**Figure S8.** Stability of sEVs isolated from the microalgae *Tetraselmis chuii* in blood plasma. Upper panel: DLS correlation functions at exemplary scattering angles of 60° of EVs (black circles), along with a forced fit (red curve) composed of the sum of the individual components, EVs and human plasma, and not additional aggregates component. Residual between fit and data are shown in the lower panel.

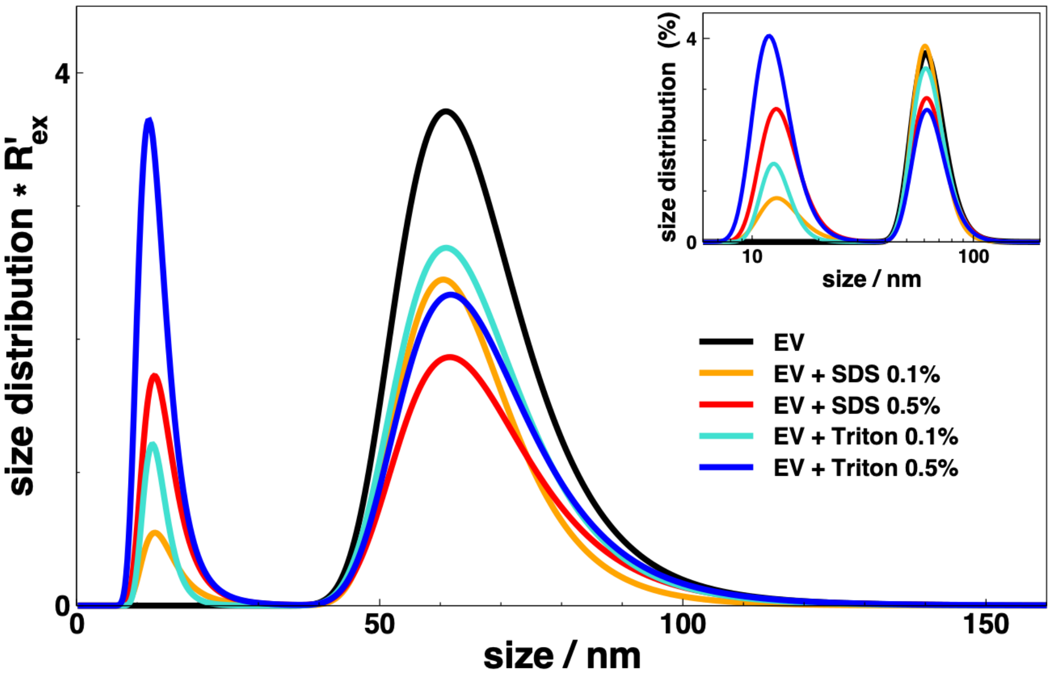


**Figure S9. Stability against detergents.** Size distribution of nanoalgosomes isolated by TFF and incubated overnight with different detergent concentrations. The distribution is normalized by the excess Rayleigh ratio ($R_{ex}$) of each sample. Inset: the distributions of the main panel in logarithmic scale and normalised to 100. The small species (around 10 nm) is related to detergent micelles. Indeed, the detergent concentration is slightly above or largely above the critical micellar concentration (cmc) for SDS or Triton, respectively. Representative measurements and analyses of *Tetraselmis chuii* nanoalgosomes (n=2).

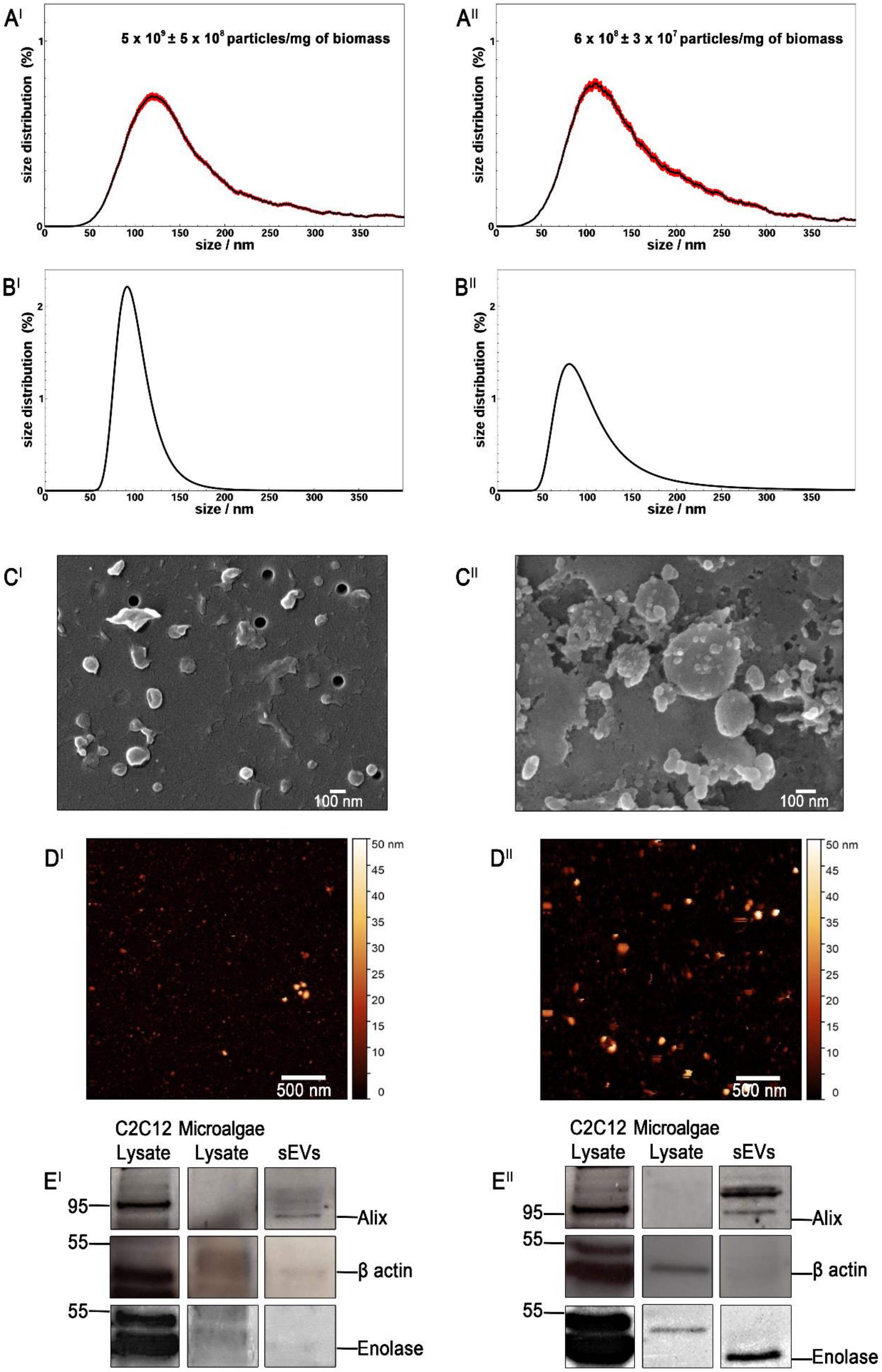


**Figure S10.** (*Continue*)

**Figure S10.** Characterization of sEVs isolated from *Dunaliella tertiolecta* ($A^{I}$-$E^{I}$) and *Amphidinium sp.* ($A^{II}$-$E^{II}$) conditioned-media. ($A^{I}$, $A^{II}$) nanoparticle tracking analysis (NTA) of sEVs (the distribution error, in red, is calculated using 5 measurements of the same sample); in insets number of sEVs/mg of dry weight biomass (results are presented by the average value ± standard deviation of the particle yield). ($B^{I}$, $B^{II}$) dynamic light scattering (DLS) analysis of sEVs. ($C^{I}$, $C^{II}$) representative images of SEM of the sEV fractions. ($D^{I}$, $D^{II}$) representative images of AFM of the sEV fractions; ($E^{I}$, $E^{II}$) a representative immunoblots of a positive control (20 μg of lysate of a mammalian cell line, C2C12), *Dunaliella tertiolecta* ($E^{I}$) and *Amphidinium sp.* ($E^{II}$) microalgae lysates (30 μg) and sEV fractions (30 μg). Representative images and data of three independent biological replicates are showed.
