# Supplementary material for: Nanoalgosomes: Introducing extracellular vesicles produced by microalgae
Source: J Extracell Vesicles. 2021 Apr 27;10(6):e12081. doi: 10.1002/jev2.12081 (PMC8077145; doi:10.1002/jev2.12081)
Supplement: Supplementary file 1 — Supporting information. [file JEV2-10-e12081-s001.zip › jev212081-sup-0002-SuppMat.pdf]

Supporting Methods

Nanoalgosomes: introducing extracellular vesicles produced by microalgae

Giorgia Adamo[1,a], David Fierli[2,a], Daniele P. Romancino[1,a], Sabrina Picciotto[1,a], Maria E. Barone[2,a], Anita Aranyos[2,a], Darja Božič[3,a], Svenja Morsbach[4,a], Samuele Raccosta[5,a], Christopher Stanly[6,a], Carolina Paganini[7,a], Meiyu Gai[4], Antonella Cusimano[1], Vincenzo Martorana[5], Rosina Noto[5], Rita Carrotta[5], Fabio Librizzi[5], Loredana Randazzo[5], Rachel Parkes[2], Umberto Capasso Palmiero[7], Estella Rao[5], Angela Paterna[5], Pamela Santonicola[6], Ales Iglič[3], Laura Corcuera[8], Annamaria Kisslinger[9], Elia Di Schiavi[6], Giovanna L. Liguori[10], Katharina Landfester[4], Veronika Kralj-Iglič[3], Paolo Arosio[7], Gabriella Pocsfalvi[6], Nicolas Touzet[2], Mauro Manno[5,b,#] and Antonella Bongiovanni[1,b,#]

[1]Institute for Research and Biomedical Innovation (IRIB) - National Research Council of Italy (CNR), Palermo, Italy
[2]Centre for Environmental Research Innovation and Sustainability Institute of Technology Sligo, Sligo, Ireland
[3]University of Ljubljana (UL), Ljubljana, Slovene
[4]Max Planck Institute for Polymer Research (MPIP), Mainz, Germany
[5]Institute of Biophysics (IBF) - National Research Council of Italy (CNR), Palermo, Italy
[6]Institute of Biosciences and BioResources (IBBR) - National Research Council of Italy (CNR), Naples, Italy
[7]Department of Chemistry and Applied Biosciences, ETH Zurich, Zurich, Switzerland
[8]Zabala Innovation Consulting, Pamplona, Spain
[9]Institute of Experimental Endocrinology and Oncology (IEOS) - National Research Council of Italy (CNR), Naples, Italy
[10]Institute of Genetics and Biophysics (IGB) - National Research Council of Italy (CNR), Naples, Italy

[a]These first Authors contributed equally to this work
[b]These last Authors contributed equally to this work

#Corresponding Authors: antonella.bongiovanni@cnr.it; https://orcid.org/0000-0002-0307-4043; mauro.manno@cnr.it; https://orcid.org/0000-0001-9843-0428

*All the listed Authors are members of the VES4US consortium (H2020 grant agreement #801338)

FLUORESCENCE CORRELATION SPECTROSCOPY (FCS)

A Di-8-ANEPPS (Sigma Aldrich) stock solution (2mM in DMSO) was diluted 1:50 in DMSO (40 μM) and filtered using a 20 nm syringe filter. An aliquot of about $5x10^{10}$ EV particles/ml was stained with 250 nM probe solution, spinned-down and incubated at 4°C overnight. FCS experiments were performed using a Hamamatsu C9413-01 instrument equipped with a 473 nm excitation source. Along with the samples, a 10nM Alexa-488 solution was used in the multi-well glass container as a calibrant for concentration and characteristic diffusion times to optimize the optical setup. The photon detection signal was routed to a USB hardware correlator (correlator.com) to measure the autocorrelation function $F(\tau)$. A large number of measurement repetitions were performed to allow for measurement rejection and statistical error assessment on the points of the autocorrelation function. Data were fitted using an adaptation of Contin [Jiří et al. 2018] for FCS experiments, according to the expression $F(\tau)=\int P(D_h)f(\tau,D_h)dD_h$, where $P(D_h)$ is the distribution of hydrodynamic diameters D_h, N is the average number density, and $f(\tau,D_h)$ is the autocorrelation function of a particle of hydrodynamic diameter D_h, which also depends on the shape of the experimental confocal volume, as reported in the literature [Ries and Schwille 2012; Montis et al. 2017].

MULTI ANGLE DYNAMIC LIGHT SCATTERING (DLS)

Scattered intensity and intensity autocorrelation function $g_2(t)$ were measured simultaneously at different scattering angle ϑ, corresponding to different scattering vector $q=4\pi n\lambda^{-1}sin(\vartheta/2)$, where n is the refractive index of the medium. Absolute values for scattered intensity, Rayleigh ratio $R(q)$, were obtained by normalisation to toluene, whose Rayleigh ratio at 532 or 632.8 nm was taken as $28x10^{-6}$ or $14\ x10^{6}\ cm^{-1}$, respectively. By applying the Siegert relation, $g_2(t)=1+\beta\ |g_1(t)|^2$, where β is an instrumental parameter, one obtains the electric field autocorrelation function $g_1(t)$ which is directly related to the diffusional dynamics of each particle in the samples: $g_1(t)=\int P(D)\exp(-Dq^2t)dD$, where D is the diffusion coefficient and $P(D)$ its distribution function. The distribution $P(D_h)$ of particle sizes, or hydrodynamic diameters D_h, is then derived by using the Stokes-Einstein relation: $D_n=k_BT/(3\pi\eta D)$, where T is the temperature, η is the medium viscosity and k_B is the Boltzmann constant. For multi angle-DLS experiments, the intensity autocorrelation functions were analysed by using cumulant analysis or Laplace inversion (CONTIN algorithm [Provencher 1982]) at CNR or MPIP, respectively. The latter analyses return an unbiased estimate of the apparent hydrodynamic diameters $D_h(q)$ and hence the z-averaged hydrodynamic diameters D_{h0}, upon extrapolation to zero scattering vector q, to take into account the hydrodynamic effects, by using the following expression: $D_h(q)^{-1}= D_{h0}^{-1}(1+k_Dq^2)$ [Schmitz 1990]. Also, the static scattered intensity is proportional to the form factor $S(q) = R(q)/R(0)$, which depends upon particle size and shape. Thus, from its q dependence, one derives the average diameter D_g, which is twice the radius of gyration, by using the following expression: $S(q)^{-1}=1+1/12\ (D_g\, q)^2$, that strictly holds for $D_g\, q<1$ (Figure 1A) [Schmitz 1990]. The distribution P(D) can be also calculated by assuming a simple shape for the distribution. Here, we initially used a Gaussian distribution, defined by two parameters, the mean and the variance. Since this was not sufficient to represent the nanoalgosome size heterogeneity, we assumed a Schultz distribution for the diffusion coefficient. The latter is a two-parameter distribution, where the variance determines the higher order cumulants. This is the minimal approach to introduce a skewness or asymmetry, and it is largely justified by the typical noise level in the experimental autocorrelation functions [Mailer et al. 2015]. This method, as described in supporting figure S2, allows for obtaining a complete distribution, with an accuracy and information content higher than the classical regularization methods based on regularization procedures, such as the CONTIN algorithm [Provencher 1981, Noto et al 2012].

REFERENCES

Jiří P, Loukotová L, Hrubý M, Štěpánek P. Distribution of Diffusion Times Determined by Fluorescence (Lifetime) Correlation Spectroscopy. Macromolecules. 2018;51 (8): 2796–2804. https://doi.org/10.1021/acs.macromol.7b02158.

Mailer A.G, Clegg P.S, Pusey P.N. Particle sizing by dynamic light scattering: non-linear cumulant analysis. J Phys: Condens Matter.2015; 27:145102. DOI: 10.1088/0953-8984/27/14/145102.

Montis C, Zendrini A, Valle F, Busatto S, Paolini L, Radeghieri A, Salvatore A, Berti D, Bergese P. Size distribution of extracellular vesicles by optical correlation techiniques. J Colloid Interface Sci. 2017;158:331-338. DOI: 10.1016/j.colsurfb.2017.06.047.

Provencher S. CONTIN: A general purpose constrained regularization program for inverting noisy linear algebraic and integral equations. Comput. Phys. Comm. 1982; 27:229-242

Ries J, Schwille P. Fluorescence Correlation Spectroscopy. BioEssays. 2012; 34:361-368. DOI: 10.1002/bies.201100111.

Schmitz K.S. An Introduction to Dynamic Light Scattering by Macromolecules. Academic Press, Inc. 1990.
