# Supplementary material for: Nanoalgosomes: Introducing extracellular vesicles produced by microalgae
Source: J Extracell Vesicles. 2021 Apr 27;10(6):e12081. doi: 10.1002/jev2.12081 (PMC8077145; doi:10.1002/jev2.12081)
Supplement: Supplementary file 1 — Supporting information. [file JEV2-10-e12081-s001.zip › jev212081-sup-0003-SuppMat.pdf]

Supporting Table

Nanoalgosomes: introducing extracellular vesicles produced by microalgae

Giorgia Adamo[1,a], David Fierli[2,a], Daniele P. Romancino[1,a], Sabrina Picciotto[1,a], Maria E. Barone[2,a], Anita Aranyos[2,a], Darja Božič[3,a], Svenja Morsbach[4,a], Samuele Raccosta[5,a], Christopher Stanly[6,a], Carolina Paganini[7,a], Meiyu Gai[4], Antonella Cusimano[1], Vincenzo Martorana[5], Rosina Noto[5], Rita Carrotta[5], Fabio Librizzi[5], Loredana Randazzo[5], Rachel Parkes[2], Umberto Capasso Palmiero[7], Estella Rao[5], Angela Paterna[5], Pamela Santonicola[6], Ales Iglič[3], Laura Corcuera[8], Annamaria Kisslinger[9], Elia Di Schiavi[6], Giovanna L. Liguori[10], Katharina Landfester[4], Veronika Kralj-Iglič[3], Paolo Arosio[7], Gabriella Pocsfalvi[6], Nicolas Touzet[2], Mauro Manno[5,b,#] and Antonella Bongiovanni[1,b,#]

[1]Institute for Research and Biomedical Innovation (IRIB) - National Research Council of Italy (CNR), Palermo, Italy

[2]Centre for Environmental Research Innovation and Sustainability Institute of Technology Sligo, Sligo, Ireland

[3]University of Ljubljana (UL), Ljubljana, Slovene

[4]Max Planck Institute for Polymer Research (MPIP), Mainz, Germany

[5]Institute of Biophysics (IBF) - National Research Council of Italy (CNR), Palermo, Italy

[6]Institute of Biosciences and BioResources (IBBR) - National Research Council of Italy (CNR), Naples, Italy

[7]Department of Chemistry and Applied Biosciences, ETH Zurich, Zurich, Switzerland

[8]Zabala Innovation Consulting, Pamplona, Spain

[9]Institute of Experimental Endocrinology and Oncology (IEOS) - National Research Council of Italy (CNR), Naples, Italy

[10]Institute of Genetics and Biophysics (IGB) - National Research Council of Italy (CNR), Naples, Italy

[a]These first Authors contributed equally to this work
[b]These last Authors contributed equally to this work

#Corresponding Authors: antonella.bongiovanni@cnr.it; https://orcid.org/0000-0002-0307-4043; mauro.manno@cnr.it; https://orcid.org/0000-0001-9843-0428

*All the listed Authors are members of the VES4US consortium (H2020 grant agreement #801338)

LIST OF MINIMAL INFORMATION FOR STUDIES OF MICROALGAL EVS
(NANOALGOSOMES)

1. SOURCE FEATURES (MICROALGAE)
**MISEV2018**
To report cultivation protocol, including initial number of cell culture, expected doubling time, frequency of medium collection.
**NANOALGOSOME checklist**
**1.(a) Microalgal strain name, cultivation protocol, and dry weight biomass**<br>**1.(b) Pigment and lipids profiling** by Chromatography methods<br>1.(c) Antioxidant activity

2. EV PURIFICATION/ENRICHMENT
**MISEV2018**
Requirement: to report all details of the method(s) for reproducibility<br>Method classification:<br>- *high recovery, low specificity*: precipitation kits, centrifugal filters, single high speed ultracentrifugation;<br>- *intermediate recovery, intermediate specificity*: size-exclusion chromatography, differential ultracentrifugation, tangential flow filtration, and membrane-affinity columns;<br>- *low recovery, high specificity*: subtypes separation by size (SEC), density (density gradient), surface composition (immuno- or other affinity isolation);<br>- *high recovery and high specificity*.

NANOALGOSOME checklist (Paganini et al., 2019)

Initial method: Standard **differential ultracentrifugation (dUC)** protocol: 10,000 x g + 118,000 x g.

Current method: **Tangential flow filtration (TFF) protocol**, iterative steps: <450nm fraction, <200nm fraction.

Further methods: gradient Ultra Centrifugation (gUC)

The current election method is TFF, since it provided a high recovery and high EV specificity, as confirmed by the size distribution (NTA and DLS analyses), lipid bilayer staining (DI-8-ANEPPS staining measured by NTA in fluorescence) and Scanning Electron Microscope analyses of the microalgal extracellular nanoparticles of the TFF fraction.

3. EV QUANTIFICATION

MISEV2018

Global quantification of EVs: number of particles, amount of proteins, lipids and if possible RNA.

NANOALGOSOME checklist

3. Biogenic nanoparticle yield (one or two among the following parameters):

- **(a) nanoparticle number**, estimated by Nanoparticle Tracking Analysis
- **(b) amount of proteins**, measured by micro-bicinchoninic (BCA) colorimetric assay
- **(c) amount of lipids**, to be estimated by Infrared Spectroscopy or other methods *(work in progress)*

Due to the reasonably high recovery and specificity of microalgal EVs obtained already by TFF, only two among the above parameters is actually required to quantify EVs preparation.

4. EV IDENTITY (SIZE)

MISEV2018

EV size and size distribution can be measured by bulk techniques (different from single molecule high-resolution imaging) including resistive pulse sensing (RPS), light scattering based techniques, such as nanoparticle tracking analysis (NTA), high resolution flow cytometry, multi-angle light scattering coupled to asymmetric flow field-flow fractionation (AF4), or fluorescence based techniques, such fluorescence correlation spectroscopy (FCS).

NANOALGOSOME checklist

4. Size and size distribution of nanoparticle preparation, measured by one or more of the following techniques:

(a) dynamic light scattering (DLS), as shown in Romancino et al., 2018, or (ii) **or multi angle DLS**

(b) nanoparticle tracking analysis (NTA), in diluted sample or:

(c) fluorescence nanoparticle tracking analysis (F-NTA), on EV labelled with fluorescent dye (e.g., a fluorescent lipid probe 4-(2-[6-(dioctylamino)-2-naphthalenyl]ethenyl)-1-(3-sulfopropyl)pyridinium (DI-8-ANEPPS) specifically anchored to the lipid bilayer,

(d) fluorescence correlation spectroscopy (FCS), in nanomolar solutions of dye labelled EVs.

These are scattering based techniques with the following actual read-out parameters: hydrodynamic radius R_h, polydispersity, R_h distribution and z-averaged radius of gyration R_g. The combined use of two of them (e.g. DLS and NTA) can help in achieving more accurate and robust results. The two fluorescence techniques can be useful to directly select labeled EVs when the sample purity is low and also to assess the presence of a lipid bilayer.

5. EV IDENTITY (morphology, shape and bilayer structure)

MISEV2018

Techniques providing images of single EVs at high resolution, e.g. electron microscopy atomic-force microscopy (AFM), or super-resolution microscopy.

Show both close-up and wide-field.

NANOALGOSOME checklist

5-I. Single vesicle morphology, determined by one or two among the following techniques:

- **(a) scanning electron microscopy (SEM)**,
- **(b) atomic force microscopy (AFM)**: it is typically less resolved than SEM but requires minor sample treatment
- **(c) cryogenic transmission electron microscopy (cryo-TEM),** morphology in physiological condition (solution structure), lipid bilayer imaging.

Cryo-EM requires high concentration and thus it not suitable for screening of large sample numbers, only to be performed for selected samples.

5-II. Shape of EVs and their bilayer.

In the case of low polydispersity a rough information about EV shape can be obtained without further sample treatment by the following bulk techniques:

- **(d) Static light scattering (SLS)**, which determines the radius of gyration R_g, and structure parameter R_g/R_h which is around 1 for hollow spheres
- **(e) Fluorescence nanoparticle tracking analysis (F-NTA)**
- **(f) Small angle X-ray scattering (SAXS)** and
- **(g) Small angle Neutron scattering (SANS)** to analyse the structure and composition of the EV bilayer.

As for cryo-EM, the latter two techniques (f, g) require a large amount of highly selected sample. Thus, they can represent a challenge to achieve an in-depth information, but should not be considered as part of required “minimal” information.

6. EV IDENTITY (PROTEIN COMPOSITION AND DENSITY)

MISEV2018

Three categories of markers must be analysed in all bulk EV preparations to demonstrate the presence of EVs (Categories 1 and 2) and assess their purity from common contaminants (Category 3). To demonstrate the presence of a lipid bilayer in the material analysed at least one transmembrane or GPI-anchored extracellular protein must be shown (Category 1). Presence of cytosolic proteins demonstrates that the analysed preparation displays the structure of lipid bilayers enclosing intracellular material (Category 2); alternatively specific fluorescent dye (e.g. calcein) can be used.

The evaluation of proteins mainly present in non-EV structures is also recommended to assess the degree of purity of the EV preparation (Category 3).

NANOALGOSOME checklist

6. (a) Immunoblot analysis of EV protein markers (transmembrane and cytosolic proteins): Tetraspanin proteins or H+/ATPase | Plasma membrane H+/ATPase (to assess the existence of a lipid bilayer, Category 1). Evolutionary highly conserved proteins as Alix, HSP70, enolase, or beta-actin (to assess the existence of intact vesicles).

6. (b) Density determination by gUC

Positive and negative controls have to be included (e.g. lysates of a mammalian cell line, lysates of the microalgal strains, small and large EV fractions).

Different antibodies specific for plant proteins or *ad hoc* designed for microalgal-conserved antigens may be used.

7. VESICLE IDENTITY (TOPOLOGY)
**MISEV2018**
Determine the topology of EV-associated components, which may be important for the function.
**NANOALGOSOME checklist**
**7.(a) Fluorescamine assay** to determine the number of accessible reactive primary NH_2 groups in EV surface. These groups are needed for surface functionalisation, and an exact quantification is useful to calculate the stoichiometry. The NH2 evaluation will also be performed by performing mild digestions.
**8. VESICLE STABILITY**
**MISEV2018**
No explicit indication is given by MISEV2018 on EV stability
**NANOALGOSOME checklist**
**8. (a) Zeta potential measurement,** to assess the surface charge of EVs; optimal surface charge would be slightly negative (not < -30 mV) to avoid potential immunoglobulin aggregation induction.<br>**8. (b) Stability test/quality control in biological fluids,** e.g. in human or animal blood plasma, to check for aggregation induced by biomolecules/physiological conditions (Raush 2010). It is performed by DLS with multicomponent analysis.<br>**8. (c) Resistance to detergents**, in order to assess membrane permeabilization and its overall resistance (to be performed by DLS or F-NTA)

9. BIOACTIVITY

MISEV2018

Recommendation: to control if a specific function is effectively EV-associate or it may be related to non-EV component (perform dose-response studies, negative controls, non-EV component separation).

NANOALGOSOME checklist

9. Cytotoxicity Assays

(a) ***In vitro large screening of EVs cytotoxicity***, using commercially available cell lines, tumorigenic and not

- MTS assay (metabolized by mitochondria) measures viability of cells;
- Brdu assay (measuring synthesized DNA) mirrors proliferation of cells;
- Acridine Orange assay (accumulating in lysosomes) allows to distinguish between Necrosis and Apoptosis.

(b) Cellular uptake

(c) In vivo EV cytotoxicity: EV neurotoxicity will be assessed on *C.elegans* models of neurodegenerative diseases

These experiments will be performed in the project to achieve more extended knowledge on EV to be used for the foreseen purposes. Due to their complexity and extension, these tasks should not be considered as simple “minimal” information.

10. PREPARATION QUALITY CONTROL (PURITY)

MISEV2018

Purity controls include proteins found in most common EV preparations from other source, such as Alix and HSP70, as well as a plasma membrane marker (i.e., H+/ATPase). These protein markers have to be present in the gUC algosome fraction corresponding to the sEV fraction with the predicted density (*e.g.,* 1.13 g/ml).

Perform functional assays after rigorous separation of EV and non-EV components. If the activity is primarily associated with EVs, the use of proper negative controls for conditioned medium without cells should not retain the activity.

NANOALGOSOME checklist

10.(a) Negative control on EV identity and preparation on culture media (without microalgae)

10.(b) EV quantification by labelling with fluorescent lipid specific dye (e.g., di-8-ANEPPS), to assess the presence of non-EV particles - see also 5.e.

10.(c) Density measurement and separation by density gradient, to assess the ratio between EVs and aggregates – see also 6b.

10.(d) Application of a tailor-made Quality Management System (QMS)

10.(e) Colorimetric NANoplasmonic (CONAN) assay, *a more precise, yet less straightforward, method to assess vesicle purity with respect to proteins (Busatto 2018).*

REFERENCES

- Busatto S, Giacomini A, Montis C, Ronca R, Bergese P. Uptake Profiles of Human Serum Exosomes by Murine and Human Tumor Cells through Combined Use of Colloidal Nanoplasmonics and Flow Cytofluorimetric Analysis. Anal Chem 2018, 90, 7855−7861
- Busatto S, Vilanilam G, Ticer T, et al. Tangential Flow Filtration for Highly Efficient Concentration of Extracellular Vesicles from Large Volumes of Fluid. Cells 2018b, 7, 273
- Clayton A, Boilard E, Buzas EI, et al. Considerations towards a roadmap for collection, handling and storage of blood extracellular vesicles. J Extracell Vesicles 2019, 8, 1647027
- EV-TRACK Consortium, van Deun J, Mestdagh P, et al. EV-TRACK: transparent reporting and centralizing knowledge in extracellular vesicle research. Nat Methods 2017, 14, 228–232
- Lešer, V.; Drobne, D.; Pipan, Ž.; Milani, M.; Tatti, F. Comparison of different preparation methods of biological samples for FIB milling and SEM investigation. Journal of Microscopy 2009, 233, 309-319, doi:10.1111/j.1365-2818.2009.03121.x
- Lotvall J, Hill AF, Hochberg F, et al. Minimal experimental requirements for definition of extracellular vesicles and their functions: a position statement from the International society for extracellular vesicles. J Extracell Vesicles 2014, 3, 26913
- Paganini C, Capasso Palmiero U, Pocsfalvi G, et al. Scalable Production and Isolation of Extracellular Vesicles: Available Sources and Lessons from Current Industrial Bioprocesses. Biotechnology Journal 2019, 1800528
- Pathan M, Fonseka P, Chitti SV, et al. Vesiclepedia 2019: a compendium of RNA, proteins, lipids and metabolites in extracellular vesicles. Nucleic Acids Research 47 (2019) D516–D519
- Rausch K, Reuter A, Fischer K, Schmidt M. Evaluation of Nanoparticle Aggregation in Human Blood Serum. Biomacromolecules 2010, 11, 2836-2839
- Romancino DP, Buffa V, Caruso S, et al. Palmitoylation is a post-translational modification of Alix regulating the membrane organization of exosome-like small extracellular vesicles Biochimica et Biophysica Acta (BBA)-General Subjects 2018, 1862, 2879-2887
- Théry C, Amigorena S, Raposo G, Clayton A. Isolation and characterization of exosomes from cell culture supernatants and biological fluids. Curr Protoc Cell Biol 2006, 22 Ch. 3, Unit 3
- Théry C, Witwer KW, Aikawa E, et al. Minimal information for studies of extracellular vesicles 2018 (MISEV2018): a position statement of the International society for extracellular vesicles and update of the MISEV2014 guidelines. J Extracell Vesicles 2018, 7, 1535750
- Urbanelli L, Buratta S, Tancini B, et al. The Role of Extracellular Vesicles in Viral Infection and Transmission. Vaccines (Basel). 2019;7(3):102. Published 2019 Aug 28. doi:10.3390/vaccines7030102
- Witwer KW, Soekmadji C, Hill AF, et al. Updating the MISEV minimal requirements for extracellular vesicle studies: building bridgestone reproducibility. J Extracell Vesicles 2017, 6, 1396823
